# metaPR^2^ : A database of eukaryotic 18S rRNA metabarcodes with an emphasis on protists

**DOI:** 10.1111/1755-0998.13674

**Published:** 2022-07-13

**Authors:** Daniel Vaulot, Clarence Wei Hung Sim, Denise Ong, Bryan Teo, Charlie Biwer, Mahwash Jamy, Adriana Lopes dos Santos

**Affiliations:** ^1^ UMR 7144, ECOMAP, CNRS Sorbonne Université, Station Biologique de Roscoff Roscoff France; ^2^ Asian School of the Environment Nanyang Technological University Singapore; ^3^ Department of Organismal Biology (Systematic Biology) Uppsala University Uppsala Sweden

**Keywords:** 18S rRNA, database, metabarcodes, PCR, protist, R, shiny

## Abstract

In recent years, metabarcoding has become the method of choice for investigating the composition and assembly of microbial eukaryotic communities. The number of environmental data sets published has increased very rapidly. Although unprocessed sequence files are often publicly available, processed data, in particular clustered sequences, are rarely available in a usable format. Clustered sequences are reported as operational taxonomic units (OTUs) with different similarity levels or more recently as amplicon sequence variants (ASVs). This hampers comparative studies between different environments and data sets, for example examining the biogeographical patterns of specific groups/species, as well analysing the genetic microdiversity within these groups. Here, we present a newly‐assembled database of processed 18S rRNA metabarcodes that are annotated with the PR^2^ reference sequence database. This database, called metaPR^2^, contains 41 data sets corresponding to more than 4000 samples and 90,000 ASVs. The database, which is accessible through both a web‐based interface (https://shiny.metapr2.org) and an R package, should prove very useful to all researchers working on protist diversity in a variety of systems.

## 1 INTRODUCTION

Protists, that is, microbial eukaryotes that are not plants, animals or fungi (Archibald et al., 2017), are one of the most dominant life forms on earth, comprising up to 80% of the total eukaryotic diversity in the environment (de Vargas et al., 2015; Mahé et al., 2017; Massana et al., 2015). Protists play key ecological roles and are involved in primary productivity, nutrient cycling and carbon sequestration. It is thus crucial to assess protist diversity and the factors that determine community composition in order to predict how protists will respond to environmental change (Cavicchioli et al., 2019). While protists have historically been more difficult to study due to their small size, the explosion of metabarcoding studies over the past 10 years has greatly expanded our knowledge of these organisms (Burki et al., 2021; Santoferrara et al., 2020).

Metabarcoding, which reveals the taxa present in an environment by amplifying and then massively sequencing a standardized genetic marker (Santoferrara, 2019; Taberlet et al., 2012), has become a very powerful and widespread approach to investigate protist diversity in a range of environments (marine, freshwater, soils, microbiomes, etc.) in recent years. By far, the most common marker used for eukaryotic microbes is the gene coding for ribosomal small subunit RNA (18S rRNA). This gene has the advantage of being universal and having well annotated reference databases such as Silva or PR^2^ (Guillou et al., 2013; Quast et al., 2013) which allow, for many protist groups, a precise taxonomic assignation. Within the 18S rRNA gene, several variable regions have been used as barcodes, in particular the V4 region located near the middle of the gene and the shorter V9 region located at its 3′ end (Burki et al., 2021; Pawlowski et al., 2012). The V4 region has most often been used in recent studies (Lopes dos Santos et al., 2021). Over the years, metabarcoding has been used to study various aspects of protist diversity. The first studies aimed to simply establish the real extent of eukaryotic diversity that was underestimated with traditional clone library approaches (e.g., Stoeck et al., 2009). In marine waters, metabarcoding studies now tackle more focused questions, for example analysing the distribution of protist groups in the ocean as a function of their size (de Vargas et al., 2015), the diversity of heterotrophic protists in the deep layers of the ocean (Giner et al., 2020; Obiol et al., 2021), detailed biogeographic distribution of specific taxa (e.g., Malviya et al., 2016; Yau et al., 2020), factors structuring marine plankton communities (Logares et al., 2020; Sommeria‐Klein et al., 2021), and the seasonal succession of taxa (e.g., Giner et al., 2019; Lambert et al., 2019). Fewer metabarcoding studies have been carried out in freshwater and soils, but that is rapidly changing with recent implementation of some large scale studies (e.g., for soils Mahé et al., 2017).

For bacteria and archaea, large metabarcoding projects using the 16S rRNA gene have been undertaken, such as the Earth Microbiome Project which encompassed more than 23,000 samples of both free‐living and host‐associated microbes, allowing inferences of global patterns of prokaryotic diversity (Thompson et al., 2017). For eukaryotes, although a few large scale sampling programs have been performed, such as *Tara* Oceans, Ocean Sampling Day (OSD) and Malaspina (de Vargas et al., 2015; Duarte, 2015; Kopf et al., 2015) for marine systems, most eukaryotic metabarcoding studies have targeted geographically restricted specific environments. Most studies that have performed analyses on the global ocean microbiota have relied on the three data sets mentioned, in particular *Tara* Oceans (e.g., Ibarbalz et al., 2019; Sommeria‐Klein et al., 2021). Many smaller‐scale metabarcoding studies have also been carried out, in particular for environments that have not been sampled by these expeditions, such as soils or freshwater lakes and rivers (Lopes dos Santos et al., 2021). Unfortunately, it is difficult to combine the data from these studies with those of the large scale expeditions for a range of reasons. First, even if the unprocessed data files containing raw reads have been deposited to GenBank SRA (sequence read archive), secondary data (e.g., clustered sequences) and metadata (e.g., sample coordinates, temperature) are rarely available, or, if available, are hard to locate since they are stored in a range of formats (DOCX, XLSX or TXT files) as supplementary files to the studies, often protected behind a pay‐wall. Clustered sequences can be provided as operational taxonomic units (OTUs) that depend on a specific similarity threshold or amplified sequence variants (ASVs, Callahan et al., 2016) that do not. OTUs clustered with different levels of similarity (e.g., 97 vs. 99%) are not directly comparable, meaning that if two studies are to be combined, clustering has to be performed de novo from the raw sequences. In contrast, ASVs from different studies can be directly compared. Secondly, taxonomic assignation is often conducted with different reference databases, such as GenBank, Silva or PR^2^ (Guillou et al., 2013; Quast et al., 2013). Some studies have tried to combine sets of samples from different environments (e.g., marine, freshwater and soil, Singer et al., 2021), but these efforts remain limited (for example, the Singer et al., 2021 study only included 122 sampling sites). The Ocean Barcode Atlas (Vernette et al., 2021) provides a web service allowing mapping of barcodes and diversity analyses. Unfortunately, at present it is restricted solely to *Tara* Oceans data sets and the taxonomy has not been updated since the publication of the original study (de Vargas et al., 2015). Thus, there is clearly a need to provide the protist research community with a reference database of metabarcodes which would allow full exploration of the available sequencing data by combining existing studies across different environments.

In this study, we introduce a database of metabarcodes (metaPR^2^) containing more than 4150 samples originating from 41 published studies, most using the V4 region of the 18S rRNA gene. The database focusses on ASVs in order for the different metabarcodes to be directly comparable. All raw sequence files were reprocessed with a pipeline based on the dada2 R package (Callahan et al., 2016). The taxonomy of the resulting ASVs was assigned using PR^2^ (Guillou et al., 2013) as a reference database. We have developed a web application available in two forms (website and R package) that allows analysis, visualization and download of the data. This database will be extended in the future with novel publicly available data sets and should prove very useful to the protist research community. In addition to introducing the database, we also provide basic statistics on the database and preliminary analyses of ASV diversity across different biomes.

## 2 MATERIALS AND METHODS

### 2.1 Data set selection and metabarcode processing

Data sets were selected from published studies (Table 1). Raw sequence files and metadata were downloaded from the NCBI SRA website (https://www.ncbi.nlm.nih.gov/Traces/study) when available or obtained directly from the investigators. Information about the study and about the samples (substrate, size fraction, etc.), as well as available metadata (geographic location, depth, date, temperature, etc.), were stored in three distinct tables in a master MySQL database, the coherence of which was checked with the R validate package (van der Loo & de Jonge, 2021). For each study, raw sequence files were processed independently de novo on the Roscoff ABIMS (Analysis and Bioinformatics for Marine Science) cluster. Primer sequences were removed with cutadapt version 2.8 (Martin, 2011) using the default parameters (maximum error rate = 10%) and the ‐g flag which removes any base upstream of the primers. Amplicon processing was performed under the R software version 3.5.1 (R Development Core Team, 2021) using the dada2 package version 1.14.0 (Callahan et al., 2016). Read quality was visualized with the plotQualityProfile function. Reads were filtered using the filterAndTrim function, adapting parameters (truncLen, minLen, truncQ, maxEE) according to overall sequence quality. Merging of the forward and reverse reads was undertaken with the mergePairs function using the default parameters (minOverlap = 12, maxMismatch = 0). Chimeras were removed using removeBimeraDenovo with default parameters. ASVs with similar sequences from different studies were merged together and identified with a unique 10 character code which corresponds to the start of the 40 character hash value of the sequence computed using the sha1 function from the R digest package. Taxonomic classification of ASVs was performed using the assignTaxonomy function from dada2 against the PR^2^ database (Guillou et al., 2013) version 4.14 (https://pr2‐database.org/). We did not threshold bootstrap values (minBoot = 0). ASV sequence, taxonomy assignment and bootstrap values, as well as abundance in each sample, were stored in tables in the same master database as the metadata. In order to limit the size of the online database, we removed ASVs that corresponded to less than 100 reads over all studies included in the database and did not consider sequences that had an assignment bootstrap value lower than 75% at the supergroup level. However, the master database contains all ASVs without any filter on their abundance or bootstrap values which will allow future evolution as the number of ASVs increases with the addition of new data sets. The total number of reads in each sample was normalized to 100 by dividing the number of reads for a given ASV in a given sample by the total number of reads in the sample multiplied by 100. In this way, read abundance could be expressed as % of total eukaryotic reads in the sample in visualizations (e.g., in maps, see below). Sequence processing scripts can be found at https://github.com/vaulot/Paper‐2021‐Vaulot‐metapr2/tree/main/R_processing.

**TABLE 1:** List of eukaryotic data sets and studies included in the metaPR2 database

ID	Name	Area	Ecosystem	Substrate	Samples	Technology	Region	Reads	ASVs	Bioproject	DOI
1	Ocean Sampling Day ‐ 2014 ‐ V4 LGC	Ocean survey	Coastal	Water	154	Illumina	V4	31,460	6557	PRJEB8682	10.1186/s13742‐015‐0066‐5
2	Ocean Sampling Day ‐ 2015 ‐ V4	Ocean survey	Coastal	Water	138	Illumina	V4	62,575	6033		10.1186/s13742‐015‐0066‐5
3	Ocean Sampling Day ‐ 2014 ‐ V4 LW	Ocean survey	Coastal	Water	29	Illumina	V4	313,694	5872		10.1186/s13742‐015‐0066‐5
5	MALINA cruise ‐ 2009	Arctic Ocean	Oceanic	Water	24	454	V4	6704	270	PRJNA202104	10.1038/ismej.2014.197
6	Central Arctic Ocean ‐ 2012	Arctic Ocean	Oceanic	Ice	8	454	V4	36,628	182	PRJEB7577	10.1080/09670262.2015.1077395
9	Nansen Basin ‐ 2012	Arctic Ocean	Oceanic	Water	17	454	V4	13,700	328	PRJEB11449	10.1371/journal.pone.0148512
11	Feldes Bay ‐ 2013	Southern Ocean	Coastal	Water	10	Illumina	V4	13,631	69	PRJNA254097	10.1007/s00300‐015‐1815‐8
16	Feldes Bay ‐ 2015	Southern Ocean	Coastal	Water	123	Illumina	V4	48,288	689	PRJNA645244	10.1038/s41598‐020‐80,568‐8
18	Feldes Bay sorted ‐ 2015	Southern Ocean	Coastal	sorted Phytoplankton	60	Illumina	V4	31,615	280	PRJNA645244	10.1038/s41598‐020‐80,568‐8
19	Gulf of Finland ‐ 2012‐2013	Baltic Sea	Coastal	Water, ice	73	Illumina	V4	71,195	933	PRJEB21047	10.3354/meps12645
20	Oslo fjord ‐ 2009‐2011	Atlantic Ocean	Coastal	Water	78	454	V4	4822	806	PRJNA497792	10.1111/jeu.12700
34	Malaspina ‐ depth profiles ‐ 2010‐2011	Ocean survey	Oceanic	Water	179	Illumina	V4	78,420	6075	PRJEB23771	10.1038/s41396‐019‐0506‐9
35	Malaspina ‐ surface ‐ 2010‐2011	Ocean survey	Oceanic	Water	119	Illumina	V4	194,174	7059	PRJEB23913	10.1186/s40168‐020‐00827‐8
36	Blanes Bay ‐ 2004‐2013	Mediterranean Sea	Coastal	Water	288	Illumina	V4	79,154	9141	PRJEB23788	10.1111/mec.14929
37	Baffin Bay ‐ 2013	Arctic Ocean	Oceanic	Water	32	Illumina	V4	36,046	518	PRJNA383398	10.1038/s41598‐018‐27,705‐6
38	White Sea ‐ 2013‐2015	Arctic Ocean	Oceanic	Ice	17	Illumina	V4	24,210	385	PRJNA368621	10.1007/s00248‐017‐1076‐x
39	ARK‐XXVII/3 cruise ‐ 2012	Arctic Ocean	Oceanic	water, ice, ice‐Algal aggregates	45	Illumina	V4	74,029	987	PRJEB23005	10.3389/fmicb.2018.01035.
40	Arctic Ocean ‐ Survey ‐ 2005‐2011	Arctic Ocean	Oceanic	Water	36	454	V4	7136	467	PRJNA243055	10.1128/AEM.02737‐14
41	ICESCAPE cruise ‐ 2010	Arctic Ocean	Oceanic	Water	23	454	V4	5799	259	PRJNA217438	10.1128/AEM.02737‐14
42	Nares Strait ‐ 2014	Arctic Ocean	Oceanic	Water	247	Illumina	V4	36,708	1533	PRJEB24314	10.3389/fmars.2019.00479
43	Gdansk Gulf ‐ 2012	Baltic Sea	Coastal	Water	35	454	V4	3461	267	PRJEB23971	10.1002/lno.11177
49	Bay of Naples ‐ 2011	Mediterranean Sea	Coastal	Water	8	Illumina	V4	213,716	2255	PRJEB24595	10.1093/femsec/fiw200
53	Biomarks project ‐ 2009	coast of Europe	Coastal	Water, sediments	120	454	V4	9416	1152	PRJEB9133	10.1016/j.cub.2014.02.050
69	Mariana Trench ‐ 2016 ‐ 1	Mariana Trench	Oceanic	Water	32	Illumina	V4	53,391	2800	PRJNA451086	10.1038/s41598‐018‐33,790‐4
70	Mariana Trench ‐ 2016 ‐ 2	Mariana Trench	Oceanic	Water	12	Illumina	V4	15,713	213	PRJNA399026	10.3389/fmicb.2018.02023
150	River Saint‐Charles ‐ 2016‐2017	Canada	freshwater Rivers	Water	142	Illumina	V4	8614	862	PRJNA486319	10.3389/fmicb.2019.02359
183	Lake Fuxian ‐ 2015	China	freshwater Lakes	Water	17	Illumina	V4	67,202	764	PRJNA534173	10.3389/fmicb.2019.02016
185	Lake Chaohu ‐ 2014‐2015	China	freshwater lakes	Water	24	Illumina	V4	63,312	999	PRJNA534176, PRJNA330896	10.1016/j.scitotenv.2019.134803
195	Lake Baikal ‐ 2013	Siberia	freshwater Lakes	Water	23	Illumina	V4	66,056	431	PRJEB24415	10.3390/microorganisms8040543
196	Lake Chevreuse ‐ 2012	France	freshwater Lakes	Water	12	454	V4	8480	124	PRJNA259710	10.1111/1462‐2920.12591
197	Lakes mountain ‐ 2013	Austria, Chile, Ethiopia	freshwater Lakes	Water	19	Illumina	V4	54,102	608	PRJNA299108	10.1111/mec.13633
198	Lake Garda	Italy	freshwater Lakes	Water	64	Illumina	V4	53,628	628	PRJEB36925	10.3389/fmicb.2020.00789
199	Soils Neotropical	Central/South America	Terrestrial	Soil	174	Illumina	V4	381,103	10,685	PRJNA317860	10.1038/s41559‐017‐0091
200	River Parana	South America	Freshwater Rivers	Water	10	Illumina	V4	137,981	1385	PRJEB23471	10.3389/fevo.2018.00099
201	Soils Swiss Alps	Switzerland	Terrestrial	Soil	580	Illumina	V4	31,824	9640	PRJEB30010	10.1111/jbi.13755
202	Lakes Argentina	Argentina	freshwater Lakes	Water	14	Illumina	V9	291,546	1648	PRJEB41211	10.1016/j.envint.2020.106262
203	Lakes Scandinavia	Scandinavia	freshwater Lakes	Water	87	454	V4	3077	301		10.1093/femsec/fiw231
204	Soils Global ‐ 2012	Global	Terrestrial	Soil	25	454	V4	1169	95		10.1038/ismej.2012.147
205	Tara Ocean ‐ V4	Ocean survey	Oceanic	Water	104	Illumina	V4	198,981	9009	PRJEB6610	10.1016/j.cell.2019.10.008
206	Tara Arctic ‐ V4	Arctic Ocean	Oceanic	Water	28	Illumina	V4	156,105	1416	PRJEB9737	10.1016/j.cell.2019.10.008
392	Tara Ocean ‐ V9	Ocean survey	Oceanic	Water	1152	Illumina	V9	695,190	30,675	PRJEB6610	10.1126/science.1261605

*Notes*: Data sets sequenced with 454 technology are single reads while those processed with Illumina are paired end reads. The column “Samples” corresponds to the number of samples that have more than 1000 reads after processing. The column “Region” correspond to the 18S rRNA gene region used for metabarcoding. The column “Reads” corresponds to mean number of reads per sample after processing. The column “ASVs” corresponds to the number of ASVs in the data set after removing ASVs that have less than 100 reads over all the data sets.

### 2.2 Metabarcode clustering

Since the data sets included in metaPR^2^ used different sets of primers (see below Table S3), for the purpose of this study we clustered ASVs with 100% similarity using the –cluster_fast option of vsearch version 2.18.0. ASVs within each cluster were merged together, using the centroid ASV as the new ASV, called cASV. This led to a slight reduction in the total number of ASVs from 79,000 to 70,000 once clustered. In general, sequences included in a given cluster were widely overlapping, although a few bases could be different outside the overlap region, indicating some microdiversity within these clusters (Figure S1). Clustering was only used in the framework of this study and the data provided in the web application are not clustered.

### 2.3 Metabarcode similarity to reference sequences

In order to evaluate the similarity of ASVs to existing reference sequences, in the context of this study we followed the approach of Metz et al. (2022). We compared ASVs to sequences from the PR^2^ database (Guillou et al., 2013) version 4.14 (https://pr2‐database.org/) using the –usearch_global option of vsearch with iddef = 2. The similarity information was stored in the MySQL database, then retrieved and merged with the ASV information using an R script. Alpha and beta diversity analyses were performed using the R phyloseq package (McMurdie & Holmes, 2013).

### 2.4 Ecological function

We used Table S1 from the study by Sommeria‐Klein et al. (2021) which assigns one of four ecological functions (phototroph, phagotroph, parasite, metazoa) to taxonomic groups (mostly at the class or division level). We merged this table with the PR^2^ taxonomy table, propagating the ecological function down to the species level. For taxonomic groups for which the Sommeria‐Klein et al. (2021) study had not defined any function, we assigned a function based on general knowledge for protists, generating a new table (Table S1).

### 2.5 Diversity analyses

Diversity analyses were performed with the phyloseq R package (McMurdie & Holmes, 2013). NMDS was based on Bray–Curtis dissimilarity. Upset plots to visualize the number of cASVs common to two or more environments were performed with the UpSetR R package.

### 2.6 R shiny application

All post‐processing was conducted with the R software. The data were extracted from the MySQL database using a custom script and stored in files using the R qs package that allows extremely fast loading of files (Travers, 2021). The data were post‐processed using the dplyr and tidyr packages. An R shiny application was developed to interact with the database using the following R packages: shiny, DT, shinyvalidate, shinyWidgets and shinycssloaders (Sali & Attali, 2020). Data were plotted using the ggplot2, treemapify, leaflet, leaftlet.minipie and plotly packages. Alpha and beta diversity analyses were performed using the phyloseq package (McMurdie & Holmes, 2013). The shiny application is available in two forms: a web‐based application (https://shiny.metapr2.org) and an R package (https://github.com/pr2database/metapr2‐shiny). The web interface runs on a Google Cloud Virtual Machine with a 20 Gb virtual disk and 4.5 Gb of memory. The R package can be installed on any computer and run off‐line.

## 3 RESULTS AND DISCUSSION

### 3.1 Overview of metaPR^2^ data sets

Forty‐one data sets are included in the current version (1.1) of the metaPR^2^ database (Table 1). We selected global oceanic data sets (OSD, Malaspina, *Tara* Oceans) that have been used in numerous publications (e.g., Giner et al., 2020; Ibarbalz et al., 2019; Tragin & Vaulot, 2018) as well as smaller data sets in particular from polar waters which have not been sampled in the global data sets. Eleven out of the 41 data sets were sequenced using the 454 technology and the rest with Illumina (mostly 2 × 250). The vast majority of the 41 data sets used the V4 region of the 18S rRNA gene which is the most commonly used metabarcode to date (Lopes dos Santos et al., 2021), with only two data sets representing the V9 region (*Tara* Oceans and Argentinian lakes, Table 1). The most common primer pairs used for V4 (Figure S2, Table S2 and S3) were those designed by Stoeck et al. (2010) and modified by Piredda et al. (2017). The V4 metabarcodes varied from 309 to 672 bp and were overlapping (Figure S2).

**FIGURE 1:**
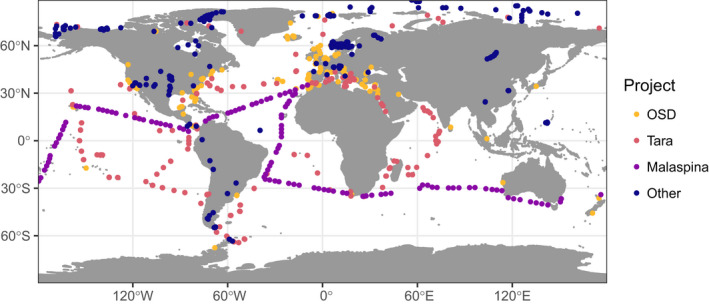
Map of stations included in the metaPR^2^ database

**FIGURE 2:**
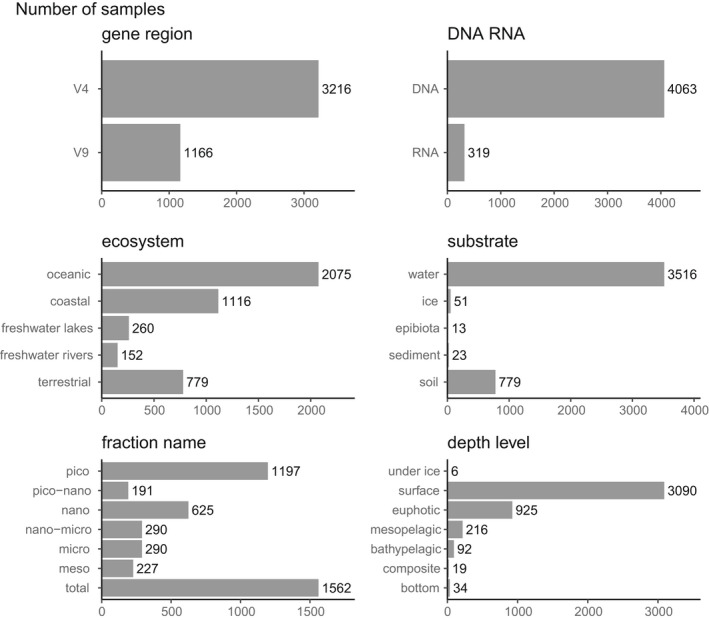
Distribution of samples by gene region, DNA or RNA, ecosystem, substrate, fraction name and depth level

The metaPR^2^ database contains more than 4150 samples (Figure 1). These samples originate from three major ecosystems: marine, freshwater and terrestrial (mostly soil substrate) (Figure 2). Among water samples, different size fractions from pico (0.2–3 *μ*m) to meso (100–1000 *μ*m) are represented, with the majority corresponding to the pico and total fractions (Figure 2). Most aquatic samples correspond to the surface or euphotic layer. Location data (longitude, latitude) are available for all samples but other metadata, for example, temperature or salinity, are missing for some samples (Figure S3).

The number of samples per data set is quite heterogeneous, ranging from less than 10 to almost 1150 for *Tara* Oceans (Table 1). The total number of reads analysed is almost 800 million for V9 and above 220 million for V4. The average number of reads per data set is also highly variable ranging from about 1000 in the older studies sequenced by 454 technology to almost 700,000 for *Tara* V9 (Table 1), which explains why overall there are more reads for V9 than V4 despite only two data sets using V9. The total number of ASVs was about 90,000. The number of ASVs in a given study ranges from less than 100 to more than 30,000 depending on both the number of samples and the depth of sequencing (Table 1). Since different studies have used different primer sets, it was necessary for the purpose of the analyses presented below to cluster ASVs with 100% similarity (cASVs, see Materials and Methods).

### 3.2 Protist composition

Overall, the database is dominated by Opisthokonta (Metazoa and fungi) and Alveolata (Dinoflagellata) (Figure S4). In this study, we decided to focus on protists and on the V4 region. The focus on protists is justified because the sampling strategy of most data sets was optimal for microbial eukaryotes. DNA from the three divisions (metazoa, plants and fungi) not included in protists were probably unevenly sampled, for example, plant seeds in soils or larval stages of metazoa in water environments. The focus on the V4 data sets that contain almost 3000 samples and 850 sites is due to that fact that the data for the V9 region are dominated by the *Tara* Oceans data set, which has been extensively analysed previously (e.g., de Vargas et al., 2015).

**FIGURE 3:**
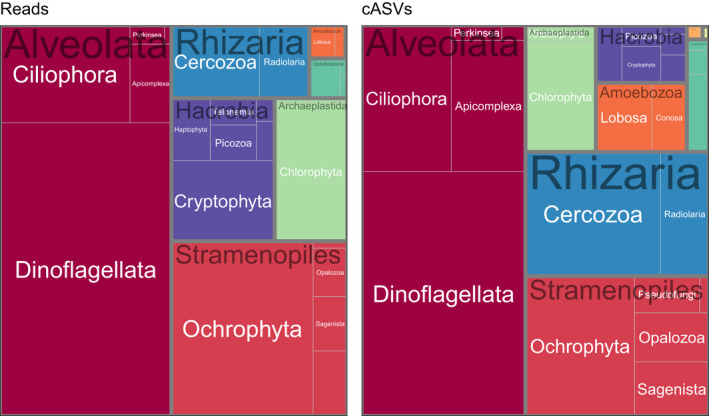
Treemaps of most abundant protist taxa (supergroup and division) for V4 data sets based on number of reads after normalization (left) or number of clustered ASVs (cASVs, right)

**FIGURE 4:**
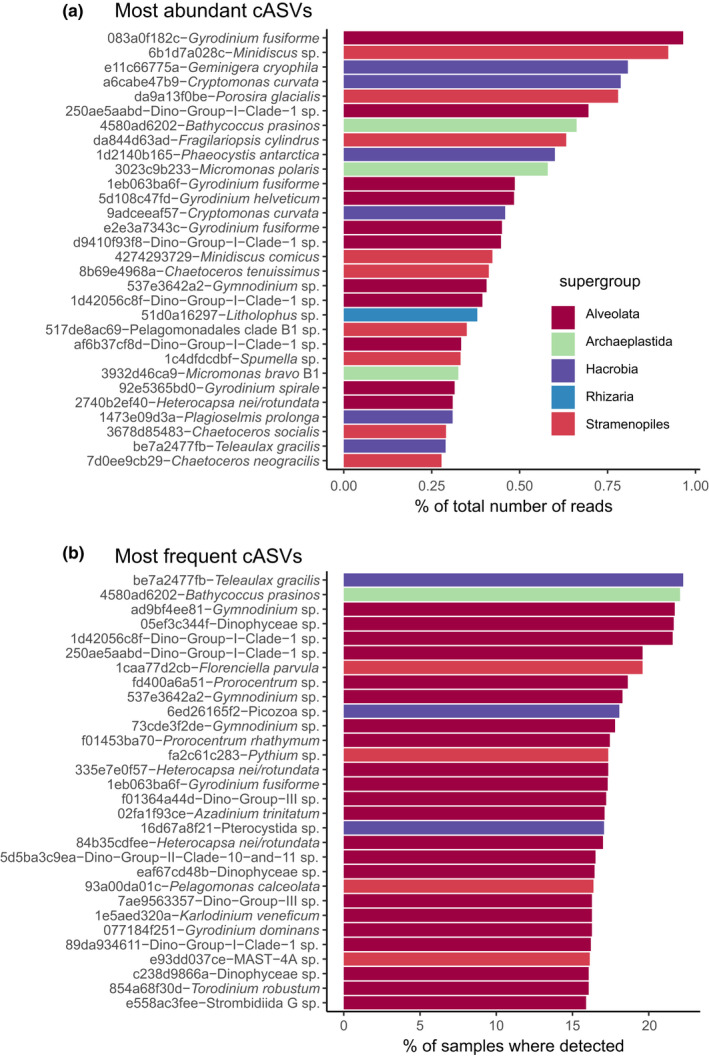
Protist V4 cASVs. (a) Most abundant cASVs (after normalization per sample). (b) Most frequent cASVs. Each cASV is coded by a 10‐letter string representing the start of the 40‐character hash value of the sequence (see Materials and Methods)

Protist sequences represent more than 41,000 ASVs (~33,000 cASVs once clustered). In terms of reads and cASVs, the database is dominated by Alveolata (in particular dinoflagellates), followed by stramenopiles (mostly photosynthetic Ochrophyta), Hacrobia, Archaeplastida and Rhizaria (Figure 3). The over‐representation of Alveolata and especially dinoflagellates in 18S rRNA‐based surveys has already been noted and is in particular due to the large number of rRNA operons per genome in this group (Zhu et al., 2005). Based on the number of cASVs, Rhizaria, despite their lower read abundance, rank just after the stramenopiles. Such a large number of unique Rhizaria sequences compared to read numbers has been observed before, possibly linked to higher error rates in regions of the RNA molecule that form secondary structures (Behnke et al., 2011). The most abundant cASVs (Figure 4a) belong to dinoflagellates.

(*Gyrodinium*), diatoms (*Minidiscus*, *Porosira*, *Fragilariopsis*), cryptophytes (*Geminigera*, *Cryptomonas*), haptophytes (*Phaeocystis*) and green algae (*Bathycoccus*, *Micromonas*). The most abundant cASVs are often also the most frequently occurring (Figure 4b and Figure S5), although for example the marine picoplanktonic genus *Florenciella* is quite frequent despite not being one of the most abundant. In contrast, the small diatom *Minidiscus* cASV is quite abundant but not present among the 30 most frequent cASVs. The contrast in read abundance and cASV frequency between these two marine phytoplanktonic genera might be a reflection of their coastal versus oceanic distribution, which can be easily observed with the online interface of metaPR^2^. *Florenciella* is a truly ubiquitous genus, found in both coastal and oceanic samples, although often in low abundance. In contrast, the nanoplanktonic diatom *Minidiscus* is mostly found in coastal environments or continental platforms, where it can form sporadic blooms (Leblanc et al., 2018). At the genus level, the five most abundant genera (Figure S6a) are the dinoflagellate *Gyrodinium*, followed by the cryptophyte *Cryptomonas*, the diatom *Chaetoceros*, the dinoflagellate *Heterocapsa* and the chlorophyte *Micromonas*. In contrast, the five most frequent genera (Figure S6b) are four dinoflagellates (*Gyrodinium*, *Prorocentrum*, *Gymnodinium* and *Heterocapsa*) followed by the diatom *Chaetoceros*. In terms of diversity, as measured by the number of cASVs belonging to a given genus (Figure S6c), three parasitic alveolates are most diverse (*Leidyana*, *Monocystis*, *Syncystis*), followed by the dinoflagellate *Prorocentrum* and the diatom *Chaetoceros*.

**FIGURE 5:**
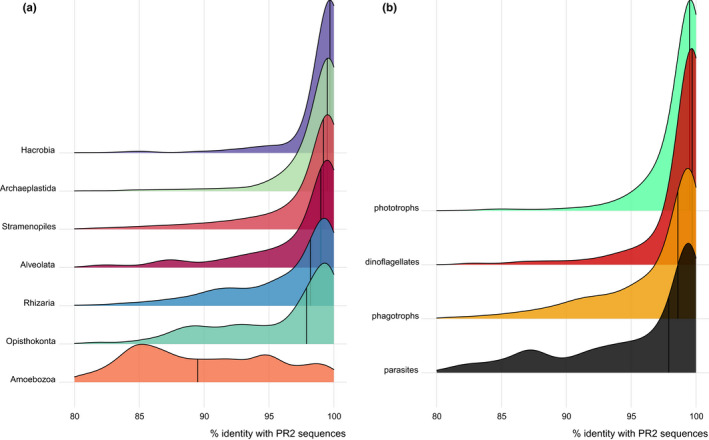
Protist V4 cASVs. Similarity of cASVs to sequences from the PR^2^ database as a (a) function of supergroup and (b) of the ecological function

Comparing the metaPR^2^ metabarcodes to reference sequences, such as those from PR^2^, reveals that there are very few novel metabarcodes for supergroups such as Hacrobia and Archaeplastida that contain many photosynthetic taxa. In contrast, for supergroups that contain mostly heterotrophic organisms, and in particular Amoebozoa, the median similarity of metabarcodes to any reference sequence is below 90% (Figure 5a) suggesting the existence of a lot of unknown taxa. A similar observation was recently reported for a restricted set of samples from a river floodplain in Argentina (Metz et al., 2022).

### 3.3 Global trends across environments

**FIGURE 6:**
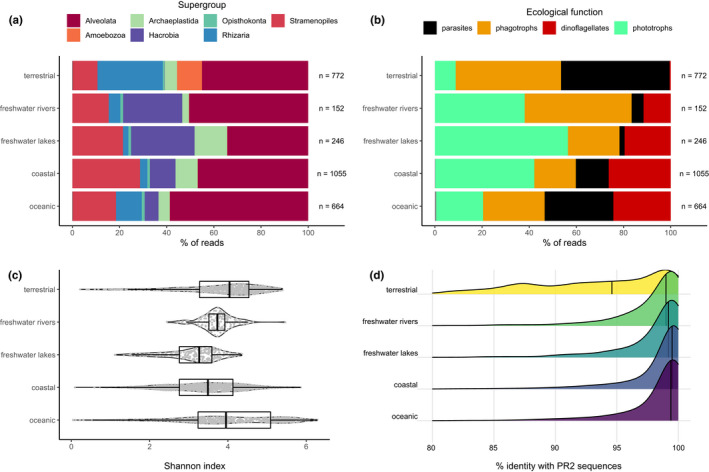
Protist V4 cASVs. Composition as a function of the environment based on (a) taxonomy or (b) on ecological function and (c) Shannon index. Similarity of cASVs to sequences from the PR^2^ database as a function of the environment (d)

**FIGURE 7:**
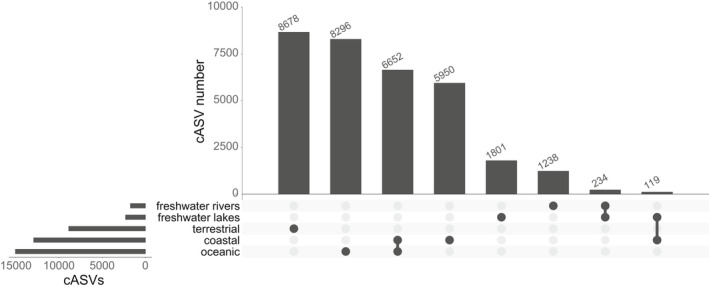
Protist V4 cASVs found on one or more environments (so‐called “upset” plot)

**FIGURE 8:**
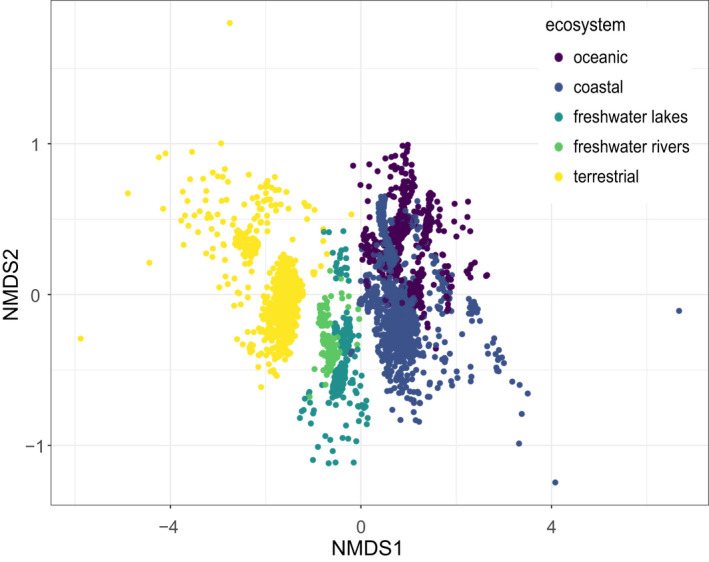
Protist V4 cASVs. NMDS analysis based on Bray–Curtis dissimilarity. Colour correspond to sample environment

Analysis of the metaPR^2^ database corroborates some trends that have been observed in studies with much fewer samples. Singer et al. (2021) examined patterns of diversity across marine, freshwater and terrestrial (soil) ecosystems based on 122 samples. Using the metaPR^2^ database which contain 23 times more samples, we were able to establish clear differences across five types of ecosystems: marine, coastal, freshwater lakes and rivers, and terrestrial (soils). In terrestrial environments, Hacrobia are almost completely absent. In contrast, Amoebozoa are absent in all environments except terrestrial ones (Figure 6a). If we use the ecological function, defined for each major taxonomic group by Sommeria‐Klein et al. (2021), the five environments clearly differ. For example, soils are characterized by the abundance of parasites, a small number of phototrophs and the absence of dinoflagellates. While parasites are abundant in soils, they are not as abundant in freshwater and increase from coastal to oceanic waters (Figure 6b). Using the Shannon index as an indicator of individual sample diversity, terrestrial ecosystems are most diverse, followed by rivers, oceanic and coastal environments, with lakes the least diverse in agreement with previous analyses (Singer et al., 2021), these differences all being significant (Figure S7). Most cASVs are restricted to a single type of ecosystem, with less than 2% (620 out of 33,235) common to two or more ecosystems if we consider coastal and oceanic ecosystems together (Figure 7). This segregation based on ecosystem type is probably not linked to the use of different primers. Since we used clustered ASVs (cASVs), we grouped together similar sequences even if they originated from data sets using different primers. Moreover, some data sets from different ecosystems used the same primer sets. For example, data sets numbers 34 and 204 (ocean), number 197 (lakes) and number 199 (soils) used the same TAReuk454FWD1/TAReukREV3 primer sets. The highest number of cASVs corresponds to marine ecosystems (coastal and oceanic), followed by terrestrial and freshwater. Interestingly, both coastal and oceanic ecosystems have a large number of specific cASVs with roughly one third purely oceanic, one third purely coastal and one third common. It is also striking that there are very few cASVs common between freshwater rivers and lakes (just above 7%). In terms of novelty, that is, of cASVs with low similarity to known sequences, terrestrial ecosystems are the least known with a median similarity below 95%, followed by rivers, lakes, coastal and oceanic ecosystems (Figure 5b). In some way, this reflects the fact that soil protists have only recently been investigated (Geisen et al., 2018). A comparison between the community structures from these different ecosystems using NMDS based on Bray‐Curtis dissimilarity (Figure 8) reveals a clear gradient: terrestrial ecosystems, followed by rivers and lakes, then coastal and oceanic ecosystems. Interestingly, river communities are the closest to soil communities, as they are probably enriched in terrestrial protists through soil drainage.

### 3.4 Shiny application

**FIGURE 9:**
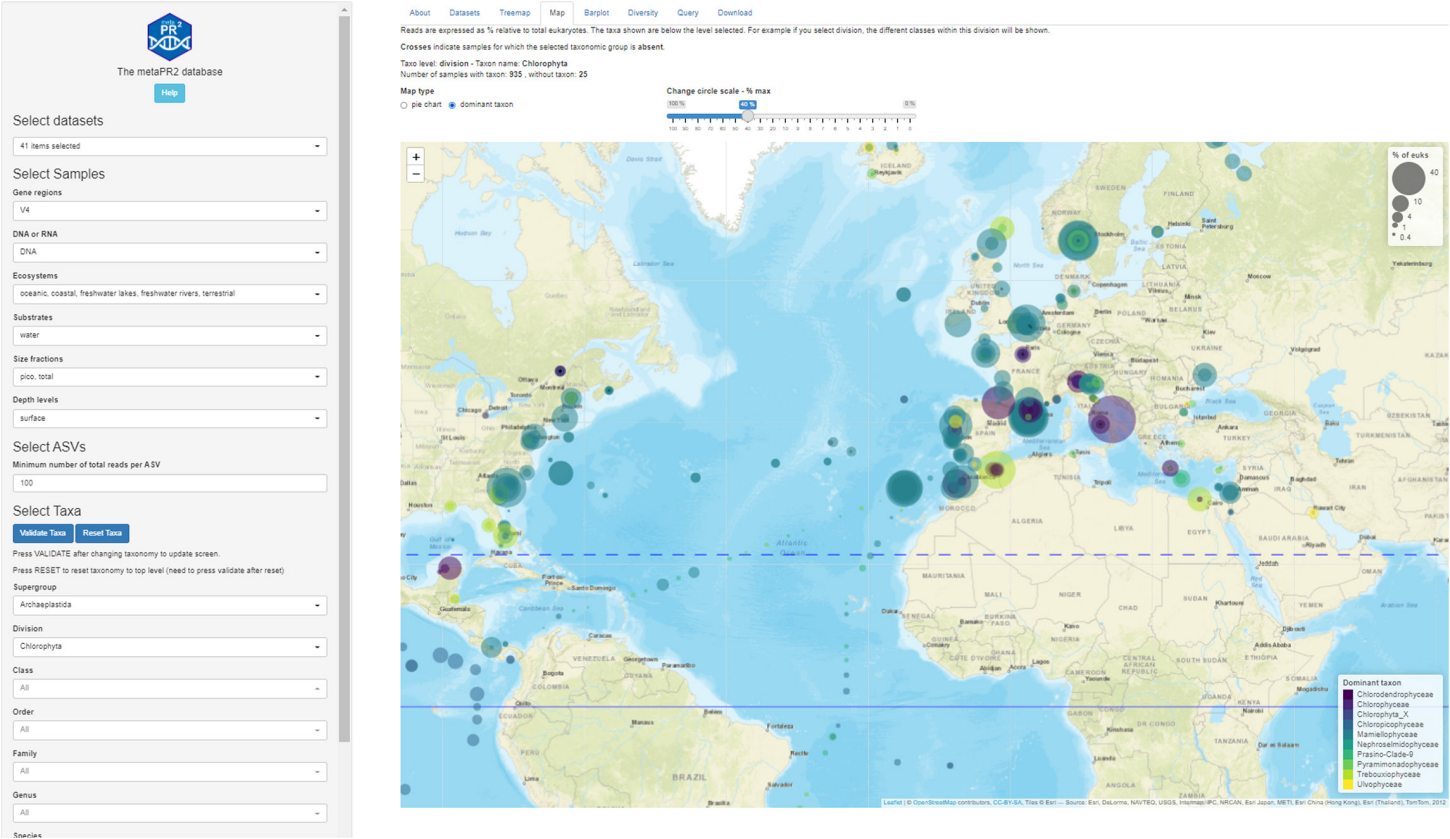
The metaPR^2^ shiny application available at https://shiny.metapr2.org

With a database of such size and complexity, it is necessary to create tools that allow to explore the database and to download the data of interest (e.g., for a specific taxonomic group or environment). We developed an R Shiny application (Figure 9 and Figure S8) for this purpose. R Shiny is an open source tool that offers numerous advantages for developing web‐based applications in comparison to coding directly under languages such as JavaScript or PHP. It offers predefined components allowing the user to interact with the data (user interface), while the server component performs the necessary computations (e.g., filtering, summarizing the data, etc.) in the background. Moreover, a Shiny application can easily be deployed on a server using open source tools such as Shiny server and can be packaged in a Docker container that can be downloaded onto a personal computer and run locally or delivered as an R package.

The metaPR^2^ Shiny application is structured in a number of panels, each dedicated to one type of analysis (e.g., map, diversity). It is possible to select/deselect specific data sets or groups of data sets, such as all oceanic data sets (Figure S9). Selection can also be based on sample characteristics such as whether samples come from DNA or RNA, the ecosystem, the type of substrate (e.g., ice, water, soil), the size fraction and the depth level (Figure S10). It is possible through reactive menus to navigate the taxonomy tree down to the species and even ASV level (potentially corresponding to cryptic species or subspecies). ASVs can be filtered based on the number of reads found for this ASV in the whole database (between 100 and 10,000). The number of total reads for a given taxonomic level can be visualized in a treemap (Figure S11) with the number of reads normalized to 100 for each sample. The distribution of any taxon can be visualized on a map (Figure S12). Two visualization modes are proposed for maps: either a pie chart at each station with a fraction of the different taxa immediately below the level selected (for example species, if genus is the level selected) or alternatively a colour circle indicating the dominant taxon immediately below the level selected (for example the dominant species in the previous example). The size of the circles is proportional to the percent of reads of the taxon selected relative to the total number of eukaryotic reads. The size of the circles can be adjusted for taxa in low abundance. Another representation is in the form of barplot (Figure S13), where the x‐axis represents the fraction of reads per taxon while the y‐axis represents one of the variables from the metadata (depth level, temperature, etc.). For continuous variables, bins are created. The barplot panel can also be used for time series with different levels of aggregation (year, month, day). Alpha and beta diversity (Figure S14) can be computed for a limited number of samples (1000 maximum). The whole set of ASV sequences can be searched using a BLAST‐like query and the resulting ASVs mapped (Figure S15). Finally, it is possible to download data sets and sample metadata as well as ASV sequences and read abundance for the data sets, samples and taxa selected (Figure S16).

Besides being very useful for research, the metaPR^2^ shiny application can also be used for teaching purposes in the field of microbial ecology. In the framework of the undergraduate course ES2304 ‐ Microbes in Natural Systems at Nanyang Technological University (Singapore), the application was used to investigate the biogeography of several groups of phytoplankton (diatoms, bolidophytes, dinoflagellates, green algae) by groups of four students in a flipped‐classroom model. Each group had to do some research on the genus it was assigned and then analyse the distribution and diversity of key species, answering questions such as whether species had ubiquitous distributions or distributions controlled by latitude or temperature and whether species appeared to contain different genotypes as reflected by the presence of several ASVs. In order to make their analysis less daunting, they only analysed the OSD, Malaspina and *Tara* Oceans V4 data sets. Despite the fact that they had only 1 week to discover the interface and produce their analyses, this hands‐on experience resulted in very positive feedback from the students, who especially enjoyed using the platform to look at “real‐world” research data.

## 4 PERSPECTIVES

Like its sister database, PR^2^, which is revised every 6–12 months with the addition of novel sequences as well as with taxonomy updates, the metaPR^2^ database will evolve with time to include more data sets and more samples, in particular from ecosystems (e.g., extreme environments), regions (e.g., tropical and southern latitudes) and substrate (e.g., host microbiomes) that are still underrepresented. We have listed more than 280 metabarcoding studies of protist diversity, for most of which data are available from GenBank SRA (Lopes dos Santos et al., 2021). These data will be processed and incorporated into the database with probably yearly releases. The taxonomy of metaPR^2^ will evolve in parallel to that of PR^2^ and we will add other functional and phenotypic traits (e.g., size, mixotrophy type) as there is clear tendency to use this approach more widely for protists (Schneider et al., 2020). We will also develop novel functionalities for the R shiny application and package, for example heat maps and phylogenetic analyses. This will constitute a very rich resource that will help researchers to compare eukaryotic communities across habitats.

## AUTHOR CONTRIBUTIONS

Daniel Vaulot conceived the study. Daniel Vaulot, Adriana Lopes dos Santos, Denise Ong, Bryan Teo, Charlie Biwer scanned the literature and metadata. Daniel Vaulot, Denise Ong, Bryan Teo, Mahwash Jamy, Charlie Biwer collected and compiled metadata from the different data sets. Daniel Vaulot developed the database structure, the analysis scripts and the R shiny application. Daniel Vaulot performed the metabarcode analyses. Clarence Wei Hung Sim compiled the functional trait information. Daniel Vaulot and Adriana Lopes dos Santos wrote the first draft of the manuscript, and all coauthors edited and approved the final version.

## CONFLICT OF INTEREST

The authors declare no competing financial interests.

### OPEN RESEARCH BADGES

This article has earned an Open Data badge for making publicly available the digitally‐shareable data necessary to reproduce the reported results. The data is available at [[insert provided URL from Open Research Disclosure Form]].

## Supporting information


Data S1

## Data Availability

The source code for the Shiny server has been made available as an R package from GitHub (https://github.com/pr2database/metapr2‐shiny, doi: 10.5281/zenodo.5992354). The source code for this study has been made available from GitHub (https://github.com/vaulot/Paper‐2021‐Vaulot‐metapr2). Source code for sequence processing has been made available from GitHub https://github.com/vaulot/Paper‐2021‐Vaulot‐metapr2/tree/main/R_processing.
