# Supplementary material for: metaPR2 : A database of eukaryotic 18S rRNA metabarcodes with an emphasis on protists
Source: Mol Ecol Resour. 2022 Jul 13;22(8):3188–201. doi: 10.1111/1755-0998.13674 (PMC9796713; doi:10.1111/1755-0998.13674)
Supplement: Supplementary file 1 — Data S1 [file MEN-22-3188-s001.pdf]

Supplementary Material for

metaPR^2: a database of eukaryotic 18S rRNA metabarcodes with an emphasis on protists.

Daniel Vaulot[1] ✉ , Clarence Wei Hung Sim[2], Denise Ong[2], Bryan Teo[2], Charlie Biwer[3], Mahwash Jamy[3], Adriana Lopes dos Santos[2]

[1] UMR 7144, ECOMAP, CNRS, Sorbonne Université, Station Biologique de Roscoff, 29680 Roscoff, France

[2] Asian School of the Environment, Nanyang Technological University, 50 Nanyang Avenue, Singapore 639798

[3] Department of Organismal Biology (Systematic Biology), Uppsala University, Uppsala, Sweden

ORCID

- Daniel Vaulot: 0000-0002-0717-5685
- Adriana Lopes dos Santos: 0000-0002-0736-4937
- Denise Ong: 0000-0001-6053-6948
- Clarence Wei Hung Sim: 0000-0003-2190-7261
- Mahwash Jamy: 0000-0002-2930-9226

✉ Corresponding author: vaulot@gmail.com

Submitted to **Molecular Ecology Resources**

Date: May 26, 2022

Keywords: 18S rRNA, metabarcodes, database, R, shiny, PCR, protists

Short title: metaPR^2 - a database of eukaryotic metabarcodes

Table S1: Ecological function of taxa according to Table S1 of Sommeria-Klein et al. (2021). Taxa present in the PR2 database for which ecological function was not present in Table S1 were assigned an ecological function based on the literature. Ecological function was propagated to all taxa below the taxon for which it was defined using an R script.

Taxon	Taxonomic level	Function	Reference
Acantharea	class	phagotrophs	Sommeria-Klein et al. 2021
Annelida	class	metazoans	Sommeria-Klein et al. 2021
Apicomplexa	class	parasites	Sommeria-Klein et al. 2021
Arthropoda	class	metazoans	Sommeria-Klein et al. 2021
Endomyxa-Ascetosporea	class	parasites	Sommeria-Klein et al. 2021
Ascomycota	class	phagotrophs	Sommeria-Klein et al. 2021
Bacillariophyta	class	phototrophs	Sommeria-Klein et al. 2021
Basidiomycota	class	phagotrophs	Sommeria-Klein et al. 2021
Bicoecea	class	phagotrophs	Sommeria-Klein et al. 2021
Bolidophyceae	class	phototrophs	Sommeria-Klein et al. 2021
Bryozoa	class	metazoans	Sommeria-Klein et al. 2021
Chaetognatha	class	metazoans	Sommeria-Klein et al. 2021
Chlorarachniophyceae	class	phototrophs	Sommeria-Klein et al. 2021
Chlorophyceae	class	phototrophs	Sommeria-Klein et al. 2021
Chloropicophyceae	class	phototrophs	Sommeria-Klein et al. 2021
Choanoflagellatea	class	phagotrophs	Sommeria-Klein et al. 2021
Chrompodellids	division	phagotrophs	Sommeria-Klein et al. 2021
Chrysophyceae	class	phototrophs	Sommeria-Klein et al. 2021
Chytridiomycota	class	parasites	Sommeria-Klein et al. 2021
Ciliophora	division	phagotrophs	Sommeria-Klein et al. 2021
Cnidaria	class	metazoans	Sommeria-Klein et al. 2021
Cryomonadida	class	phagotrophs	Sommeria-Klein et al. 2021
Cryptophyta	division	phototrophs	Sommeria-Klein et al. 2021
Ctenophora	class	metazoans	Sommeria-Klein et al. 2021
Dactylopodida	order	parasites	Sommeria-Klein et al. 2021
Dictyochophyceae	class	phototrophs	Sommeria-Klein et al. 2021
Dinophyceae	class	dinoflagellates	Sommeria-Klein et al. 2021
Diplonemida	order	phagotrophs	Sommeria-Klein et al. 2021
Ebriida	order	phagotrophs	Sommeria-Klein et al. 2021
Echinodermata	class	metazoans	Sommeria-Klein et al. 2021
Eucyrtidium	genus	phagotrophs	Sommeria-Klein et al. 2021
Euglenida	class	phagotrophs	Sommeria-Klein et al. 2021

Table S1: *(continued)*

Taxon	Taxonomic level	Function	Reference
Foraminifera	division	phagotrophs	Sommeria-Klein et al. 2021
Haptophyta	division	phototrophs	Sommeria-Klein et al. 2021
Katablepharidophyta	division	phagotrophs	Sommeria-Klein et al. 2021
Kinetoplastea	class	parasites	Sommeria-Klein et al. 2021
Labyrinthulomycetes	class	phagotrophs	Sommeria-Klein et al. 2021
Dino-Group-I	order	parasites	Sommeria-Klein et al. 2021
Dino-Group-II	order	parasites	Sommeria-Klein et al. 2021
Dino-Group-III	order	parasites	Sommeria-Klein et al. 2021
Dino-Group-IV	order	parasites	Sommeria-Klein et al. 2021
Dino-Group-V	order	parasites	Sommeria-Klein et al. 2021
Mamiellophyceae	class	phototrophs	Sommeria-Klein et al. 2021
MAST-1	class	phagotrophs	Sommeria-Klein et al. 2021
MAST-10	class	phagotrophs	Sommeria-Klein et al. 2021
MAST-11	class	phagotrophs	Sommeria-Klein et al. 2021
MAST-12	class	phagotrophs	Sommeria-Klein et al. 2021
MAST-3	class	phagotrophs	Sommeria-Klein et al. 2021
MAST-4	class	phagotrophs	Sommeria-Klein et al. 2021
MAST-6	class	phagotrophs	Sommeria-Klein et al. 2021
MAST-7	class	phagotrophs	Sommeria-Klein et al. 2021
MAST-8	class	phagotrophs	Sommeria-Klein et al. 2021
MAST-9	class	phagotrophs	Sommeria-Klein et al. 2021
Mesomycetozoa	division	parasites	Sommeria-Klein et al. 2021
MOCH-1	class	phototrophs	Sommeria-Klein et al. 2021
MOCH-2	class	phototrophs	Sommeria-Klein et al. 2021
Mollusca	class	metazoans	Sommeria-Klein et al. 2021
Nassellaria	order	phagotrophs	Sommeria-Klein et al. 2021
Nemertea	class	metazoans	Sommeria-Klein et al. 2021
Oomycota	class	parasites	Sommeria-Klein et al. 2021
Pelagophyceae	class	phototrophs	Sommeria-Klein et al. 2021
Platyhelminthes	class	metazoans	Sommeria-Klein et al. 2021
Porifera	class	metazoans	Sommeria-Klein et al. 2021
Pyramimonadophyceae	class	phototrophs	Sommeria-Klein et al. 2021
RAD-A	class	phagotrophs	Sommeria-Klein et al. 2021
RAD-B	class	phagotrophs	Sommeria-Klein et al. 2021
RAD-C	class	phagotrophs	Sommeria-Klein et al. 2021

Table S1: *(continued)*

Taxon	Taxonomic level	Function	Reference
Rhodophyta	division	phototrophs	Sommeria-Klein et al. 2021
Spumellaria	order	phagotrophs	Sommeria-Klein et al. 2021
Streptophyta	division	phototrophs	Sommeria-Klein et al. 2021
Telonemia	division	phagotrophs	Sommeria-Klein et al. 2021
Trebouxiophyceae	class	phototrophs	Sommeria-Klein et al. 2021
Vannellida	order	phagotrophs	Sommeria-Klein et al. 2021
Amoebozoa	supergroup	parasites	Literature
Perkinsea	division	parasites	Literature
Alveolata_X	division	parasites	Literature
Dinoflagellata	division	phagotrophs	Literature
Apusozoa	supergroup	phagotrophs	Literature
Chlorophyta	division	phototrophs	Literature
Glaucophyta	division	phototrophs	Literature
Prasinodermophyta	division	phototrophs	Literature
Centroheliozoa	division	phagotrophs	Literature
Metamonada	division	parasites	Literature
Picozoa	division	phagotrophs	Literature
Choanoflagellida	division	phagotrophs	Literature
Fungi	division	parasites	Literature
Metazoa	division	metazoans	Literature
Cercozoa	division	phagotrophs	Literature
Aurearenophyceae	class	phototrophs	Literature
Chrysomerophyceae	class	phototrophs	Literature
Eustigmatophyceae	class	phototrophs	Literature
MOCH-3	class	phototrophs	Literature
MOCH-4	class	phototrophs	Literature
MOCH-5	class	phototrophs	Literature
Phaeophyceae	class	phototrophs	Literature
Ochrophyta	division	phototrophs	Literature
Pinguiophyceae	class	phototrophs	Literature
Raphidophyceae	class	phototrophs	Literature
Synchromophyceae	class	phototrophs	Literature
Synurophyceae	class	phototrophs	Literature
Xanthophyceae	class	phototrophs	Literature
MAST-16	class	phagotrophs	Literature

Table S1: *(continued)*

Taxon	Taxonomic level	Function	Reference
MAST-22	class	phagotrophs	Literature
MAST-24	class	phagotrophs	Literature
Opalozoa	division	parasites	Literature
Pseudofungi	division	parasites	Literature
Sagenista	division	phagotrophs	Literature
Stramenopiles	supergroup	phagotrophs	Literature
Discoba	division	parasites	Literature
Archaeplastida	supergroup	phototrophs	Literature
Eukaryota_X	supergroup	parasites	Literature
Malawimonadidae	division	parasites	Literature
Radiolaria	division	phagotrophs	Literature
Protalveolata	supergroup	phagotrophs	Literature

Table S2: Eukaryotic 18S rRNA primers used for metaPR2 datasets with the number of datasets (N) where used (Table 1).

Name	Sequence	Region	Direction	Reference	DOI	N
TAReuk454FWD1	CCAGCASCYGCGGTAATTCC	V4	fwd	Stoeck et al (2010)	10.1111/j.1365-294X.2009.04480.x	21
E572F	CYGCGGTAATTCCAGCTC	V4	fwd	Comeau et al. (2011)	10.1371/journal.pone.0027492	7
3NDf	GGCAAGTCTGGTGCCAG	V4	fwd	Cavalier-Smith et al. (2009)	10.1016/j.protis.2009.03.003	2
528F	GCGGTAATTCCAGCTCCAA	V4	fwd	Cheung et al. (2010)	10.1038/ismej.2010.26	2
NSF573	CGCGGTAATTCCAGCTCCA	V4	fwd	Mangot et al. (2013)	10.1111/1462-2920.12065	2
1380F	CCCTGCCHTTTGTACACAC	V9	fwd	Amaral Zettler et al (2009)	10.1371/journal.pone.0006372	1
1389F	TTGTACACACCGCCC	V9	fwd	Amaral Zettler et al (2009)	10.1371/journal.pone.0006372	1
515F	GTGCCAGCMGCCGCGGTAA	V4	fwd	Parfrey et al. (2014)	10.3389/fmicb.2014.00298	1
528F	CCGCGGTAATTCCAGCTC	V4	fwd	Zhu et al. (2005)	10.1016/j.femsec.2004.10.006	1
EK-565F	GCAGTTAAAAAGCTCGTAGT	V4	fwd	Simon et al. (2015)	10.1111/1462-2920.12591	1
EuF-V4	CCAGCASCCGCGGTAATWCC	V4	fwd	Boscaro et al. (2017)	10.1007/s00248-016-0912-8	1
EuF-V4	CCAGCASCCGCGGTAATWCC	V4	fwd	Belevich et al. (2017)	10.1007/s00248-017-1076-x	1
TAReukFWD1	CCAGCASCYGCGGTAAT	V4	fwd	Annenkova et al. (2020)	10.3390/microorganisms8040543	1
TAReukREV3	ACTTTCGTTCTTGATYRA	V4	rev	Stoeck et al (2010)	10.1111/j.1365-294X.2009.04480.x	15
V4 18S Next.Rev	ACTTTCGTTCTTGATYRATGA	V4	rev	Piredda et al. (2017)	10.1093/femsec/fiw200	7
E1009R	AYGGTATCTRATCRTCTTYG	V4	rev	Comeau et al. (2011)	10.1371/journal.pone.0027492	6
1055R	ACGGCCATGCACCACCACCCAT	V4	rev	Alves-de-Souza et al (2011)	10.5194/bg-8-2125-2011	2
1510R	CCTTCYGCAGGTTCACCTAC	V9	rev	Lopez-Garcia et al. (2003)	10.1073/pnas.0235779100	2
NSR951	TTGGYRAATGCTTTCGC	V4	rev	Mangot et al. (2013)	10.1111/1462-2920.12065	2
V4_euk_R2	ACGGTATCTRATCRTCTTCG	V4	rev	Brate et al. (2010)	10.1038/ismej.2010.39	2
1119r	GGTGCCCTTCCGTCA	V4	rev	Parfrey et al. (2014)	10.3389/fmicb.2014.00298	1
897R	TCYDAGAATTYCACCTCT	V4	rev	Hugerth et al. (2014)	10.1371/journal.pone.0095567	1
EUK1134-R	TTTAAGTTTCAGCCTTGCG	V4	rev	Carnegie et al. (2003)	10.3354/dao054219	1
Nex_18S_0964_R	GATCCCYYAACTTTCGTTCTTGA	V4	rev	Kim et al. (2016)	10.1111/1462-2920.13523	1
picoR2	AKCCCCYAACTTTCGTTCTTGAT	V4	rev	Belevich et al. (2017)	10.1007/s00248-017-1076-x	1

Table S3: 18S rRNA primer sets used for metaPR2 datasets with the number of datasets (N) where used (Table 1). Refer to Table S2 for sequence and reference of primers.

Primer fwd	Primer rev	Region	N
TAReuk454FWD1	TAReukREV3	V4	14
TAReuk454FWD1	V4 18S Next.Rev	V4	7
E572F	E1009R	V4	6
3NDF	V4_euk_R2	V4	2
528F	1055R	V4	2
NSF573	NSR951	V4	2
1380F	1510R	V9	1
1389F	1510R	V9	1
E572F	897R	V4	1
EK-565F	UNonMet	V4	1
EuF-V4	picoR2	V4	1
F515	R119	V4	1
Nex_18S_0587_F	Nex_18S_0964_R	V4	1
TAReukFWD1	TAReukREV3	V4	1

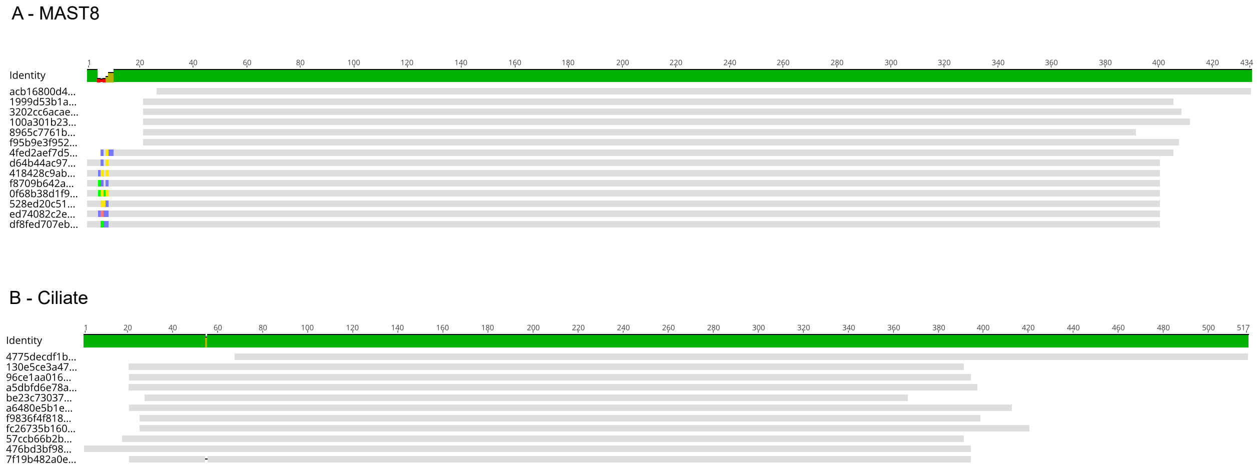

Figure S1: Two examples of V4 sequence clusters ASV (cASV) for Stramenopiles MAST 8 (A) and ciliates (B).

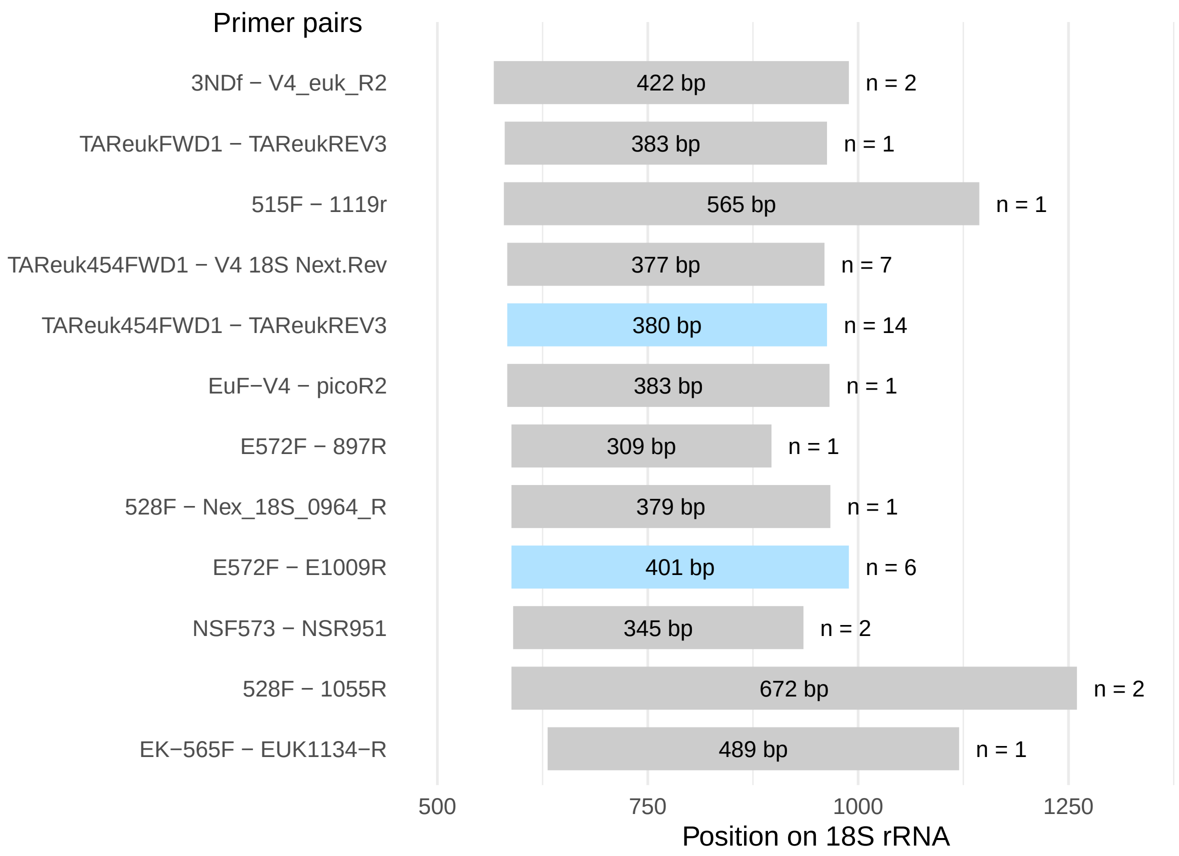

Figure S2: Amplicon size and position on the 18S rRNA gene (yeast), with the number of datasets for each V4 primer pair on the right side.

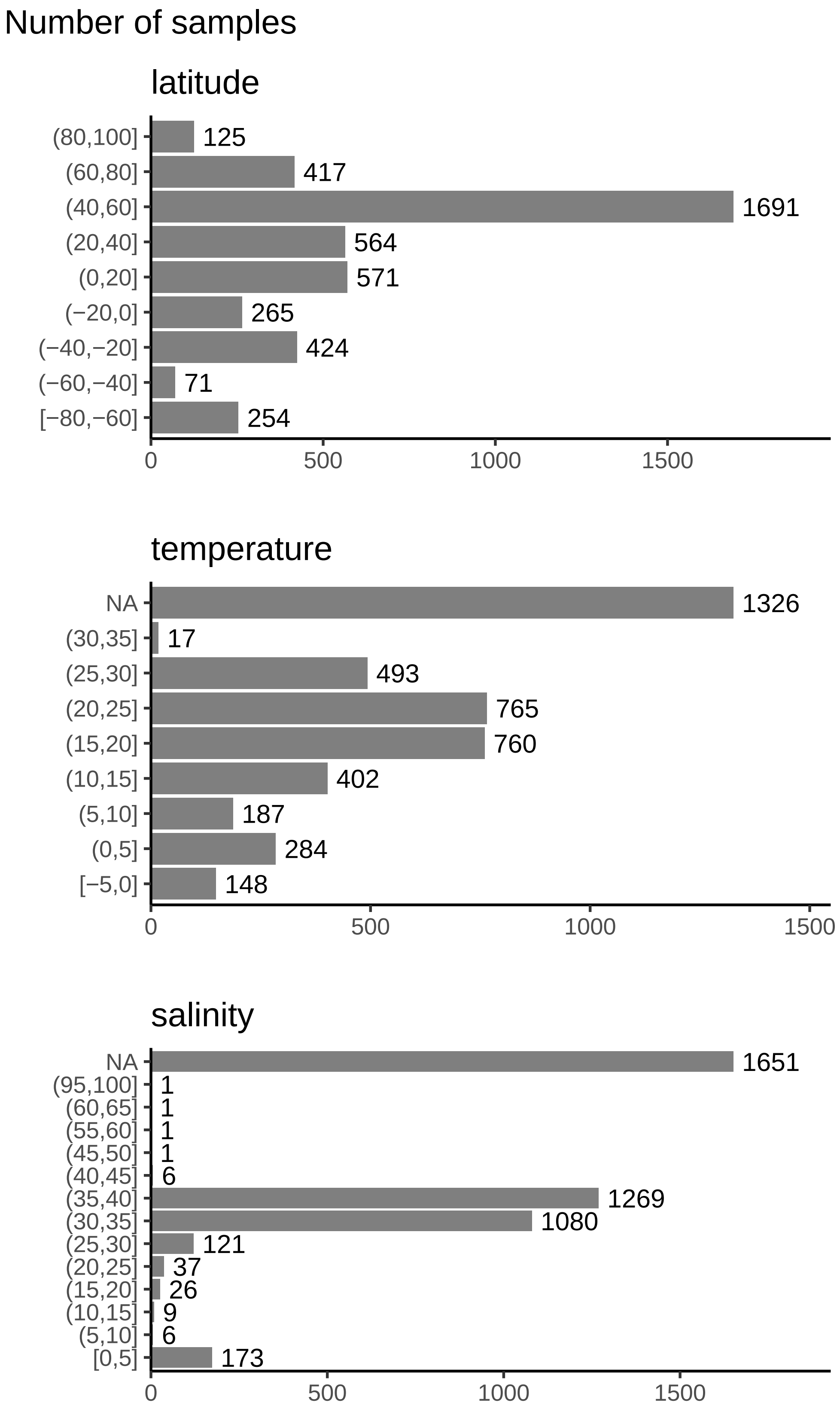

Figure S3: Distribution of samples by latitude, temperature and salinity ranges. NA corresponds to samples for which the data are not available.

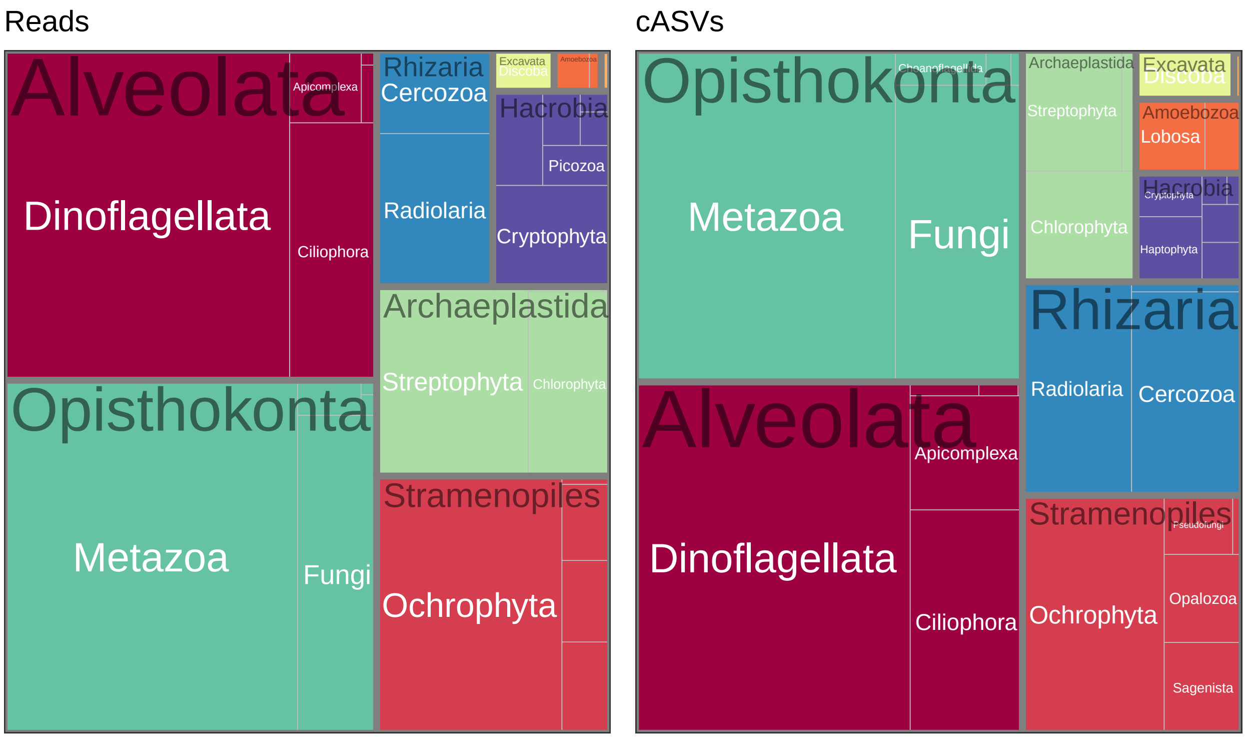

Figure S4: Treemaps of most abundant taxa (supergroup and division) for all datasets (V4 and V9) based on number of reads after normalization (left) or number of cASVs (right).

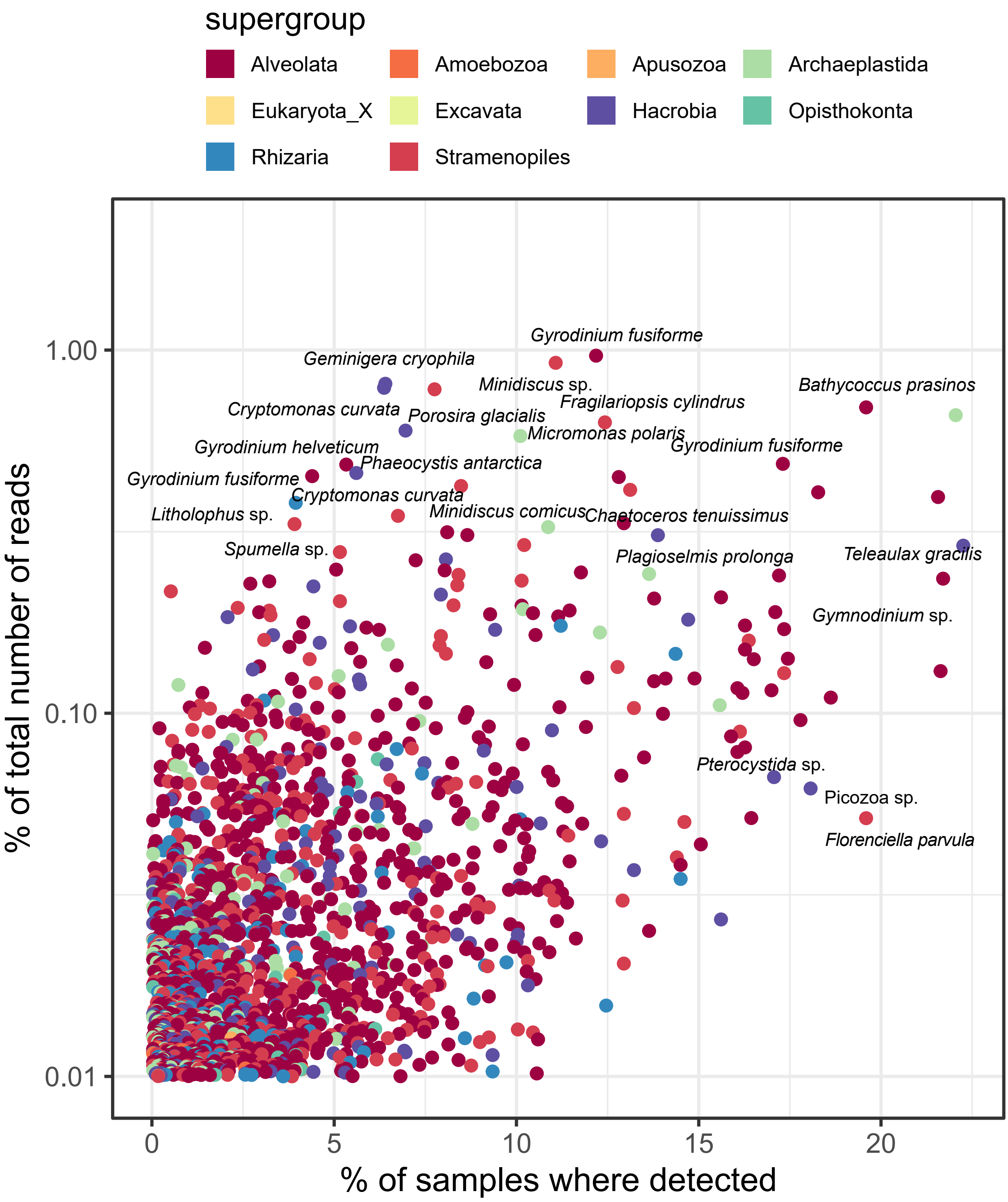

Figure S5: Protist V4 cASVs. Relationships between frequency and abundance of cASVs. Colours correspond to supergroups.

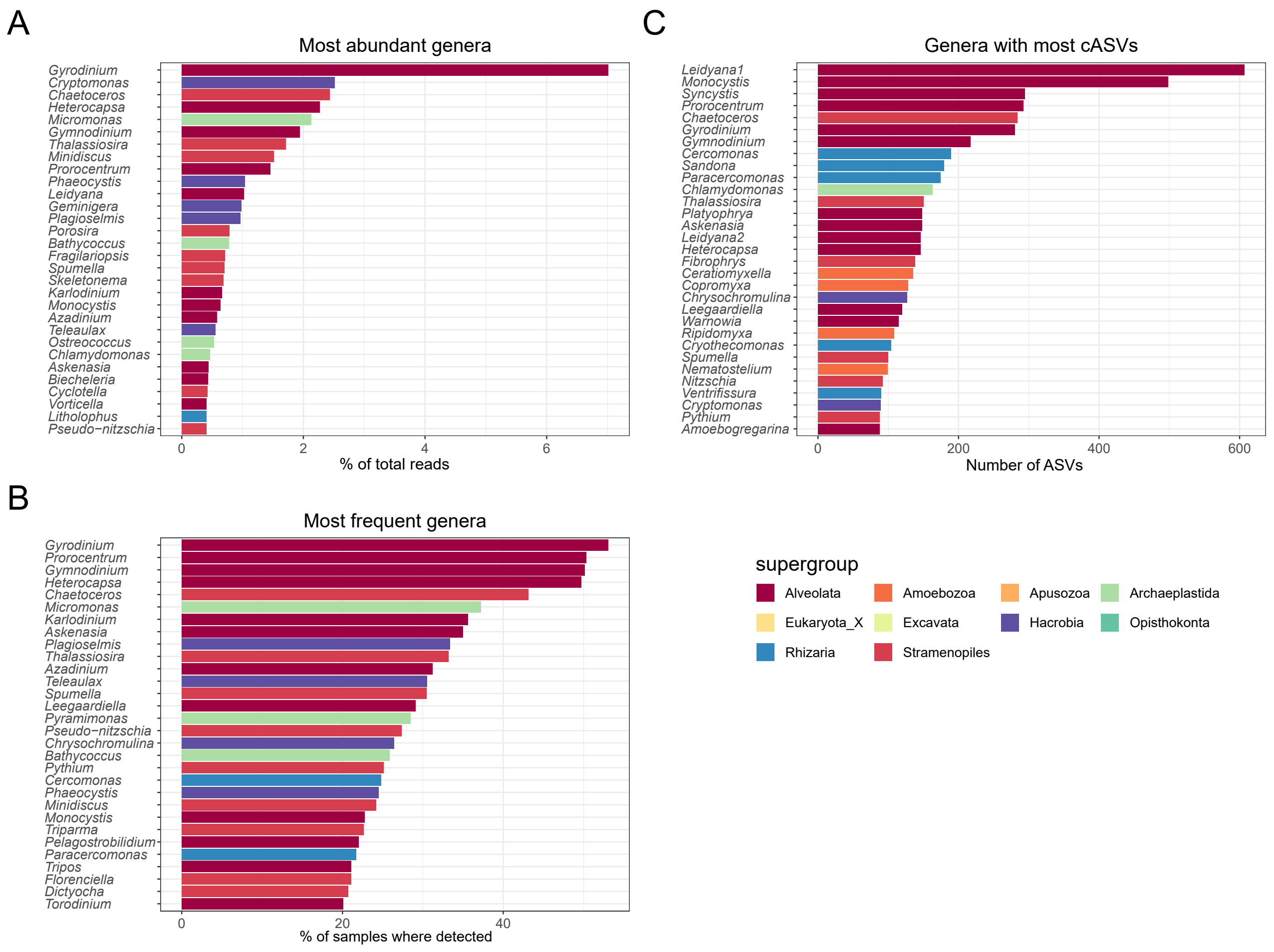

Figure S6: Protist genus analysis for the V4 dataset after normalization. A. Most abundant genera. B. Most frequent genera. C. Genera with most cASVs.

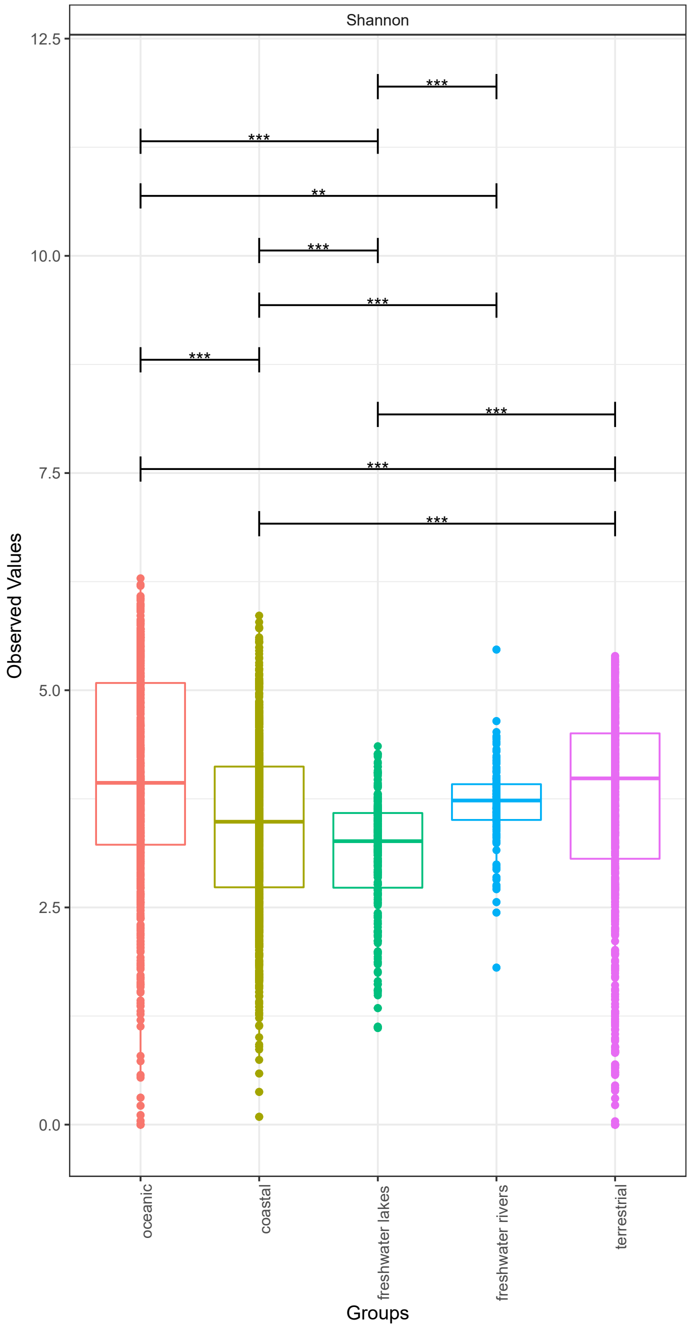

Figure S7: Protist V4 ASVs. Shannon's diversity index as a function of the environment with significance (** p-value < 0.01, *** p-value < 0.001).

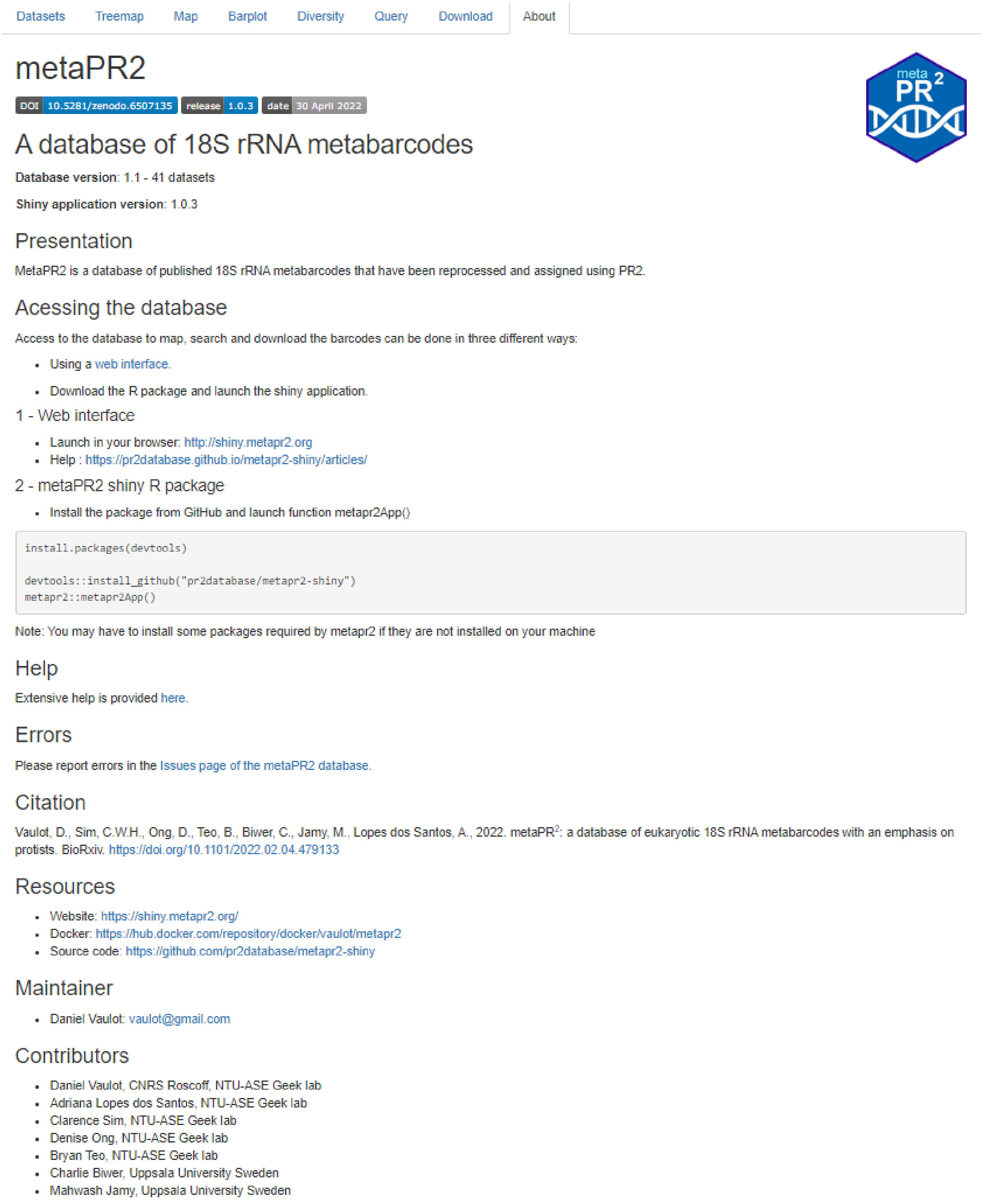

Datasets Treemap Map Barplot Diversity Query Download About

metaPR2

DOI 10.5281/zenodo.6507135 release 1.0.3 date 30 April 2022

A database of 18S rRNA metabarcodes

Database version: 1.1 - 41 datasets

Shiny application version: 1.0.3

Presentation

MetaPR2 is a database of published 18S rRNA metabarcodes that have been reprocessed and assigned using PR2.

Acessing the database

Access to the database to map, search and download the barcodes can be done in three different ways:

- Using a web interface.
- Download the R package and launch the shiny application.

1 - Web interface

- Launch in your browser: http://shiny.metapr2.org
- Help : https://pr2database.github.io/metapr2-shiny/articles/

2 - metaPR2 shiny R package

- Install the package from GitHub and launch function metapr2App()

```
install.packages(devtools)

devtools::install_github("pr2database/metapr2-shiny")
metapr2::metapr2App()
```

Note: You may have to install some packages required by metapr2 if they are not installed on your machine

Help

Extensive help is provided here.

Errors

Please report errors in the Issues page of the metaPR2 database.

Citation

Vaulot, D., Sim, C.W.H., Ong, D., Teo, B., Biwer, C., Jamy, M., Lopes dos Santos, A., 2022. metaPR^2: a database of eukaryotic 18S rRNA metabarcodes with an emphasis on protists. BioRxiv. https://doi.org/10.1101/2022.02.04.479133

Resources

- Website: https://shiny.metapr2.org/
- Docker: https://hub.docker.com/repository/docker/vaulot/metapr2
- Source code: https://github.com/pr2database/metapr2-shiny

Maintainer

- Daniel Vaulot: vaulot@gmail.com

Contributors

- Daniel Vaulot, CNRS Roscoff, NTU-ASE Geek lab
- Adriana Lopes dos Santos, NTU-ASE Geek lab
- Clarence Sim, NTU-ASE Geek lab
- Denise Ong, NTU-ASE Geek lab
- Bryan Teo, NTU-ASE Geek lab
- Charlie Biwer, Uppsala University Sweden
- Mahwash Jamy, Uppsala University Sweden

Figure S8: Shiny panel "About".

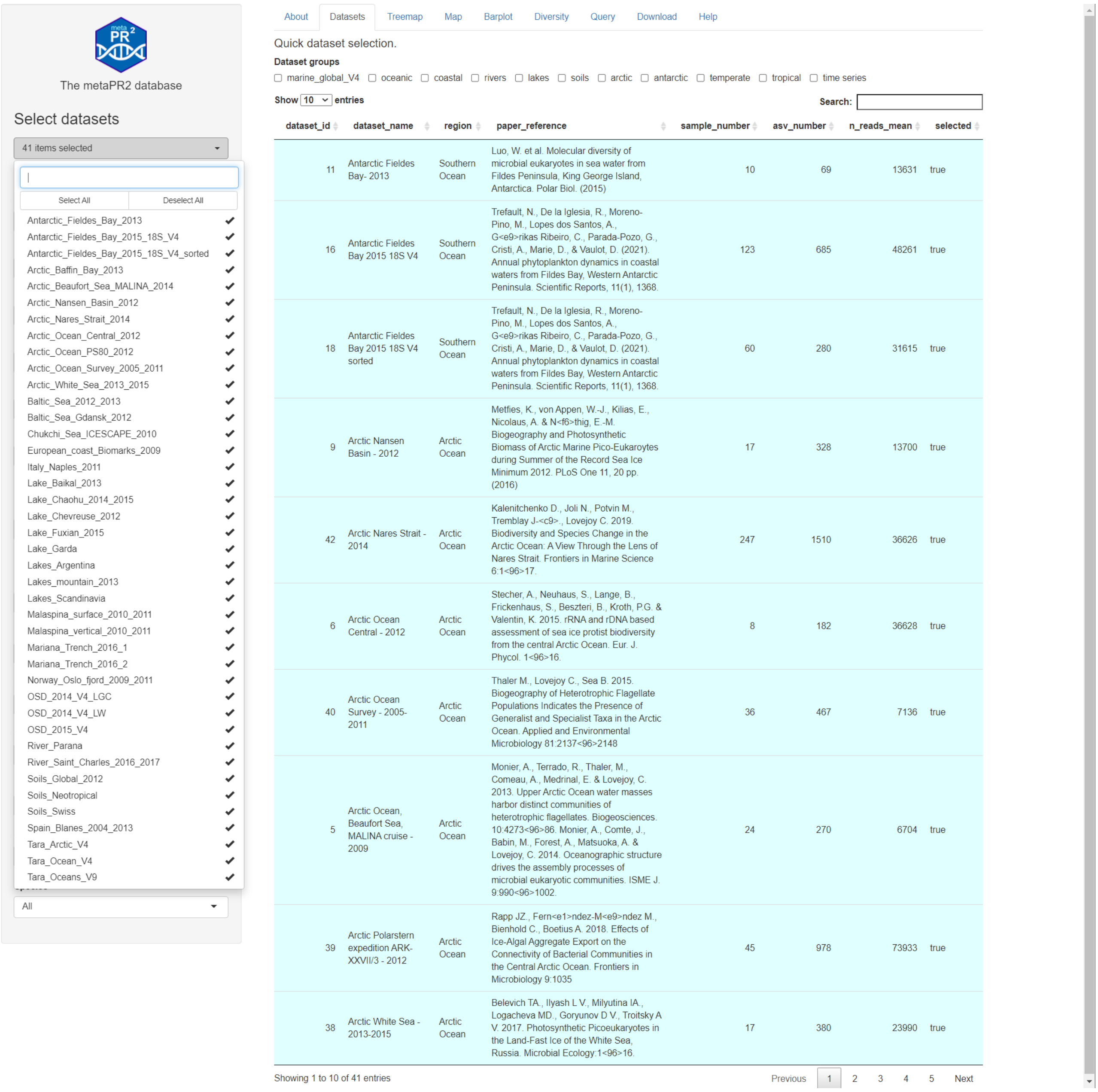

Figure S9: Shiny panel "Datasets".

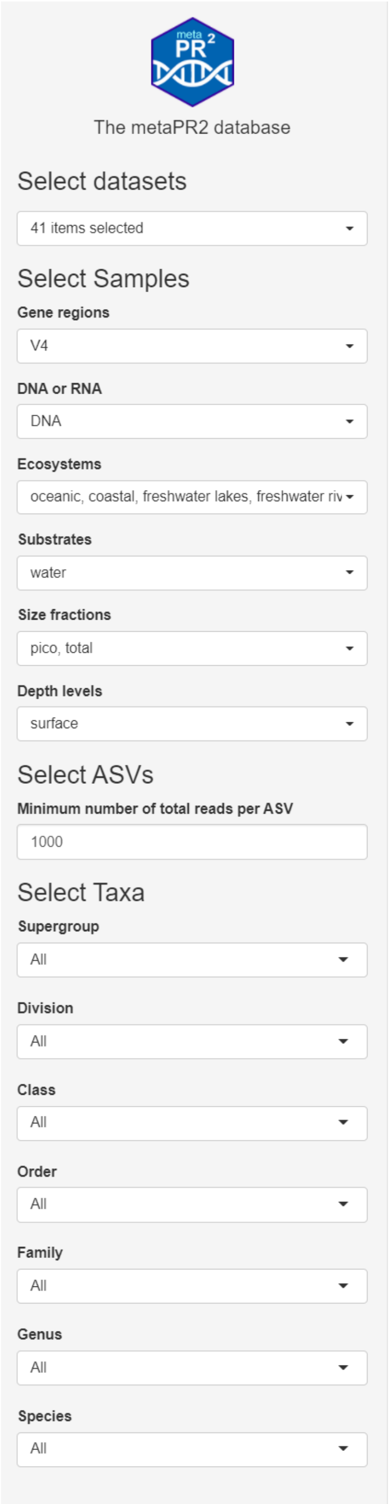

Figure S10: Shiny sample and taxonomy selection sidebar.

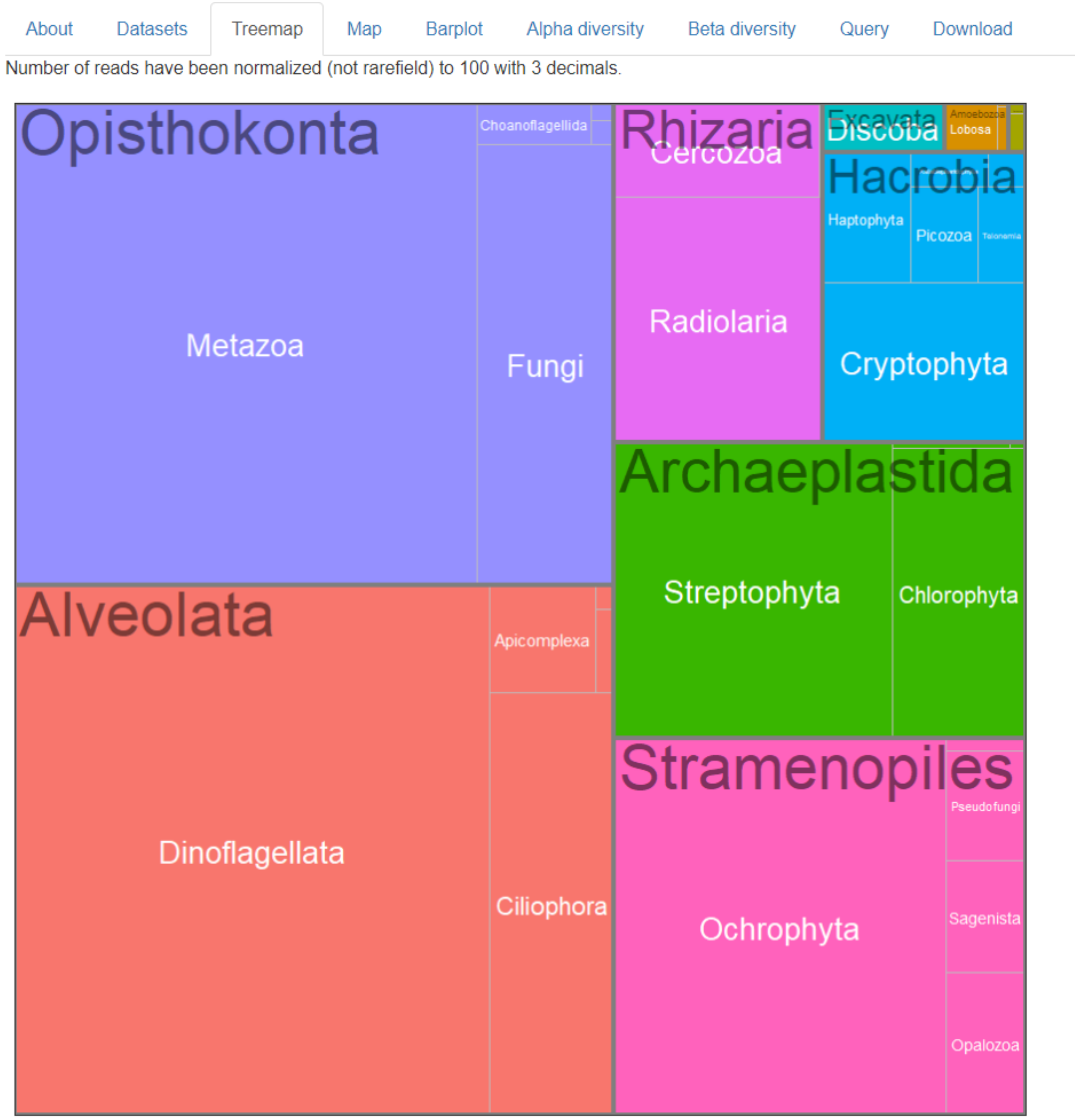

Figure S11: Shiny panel "Treemap".

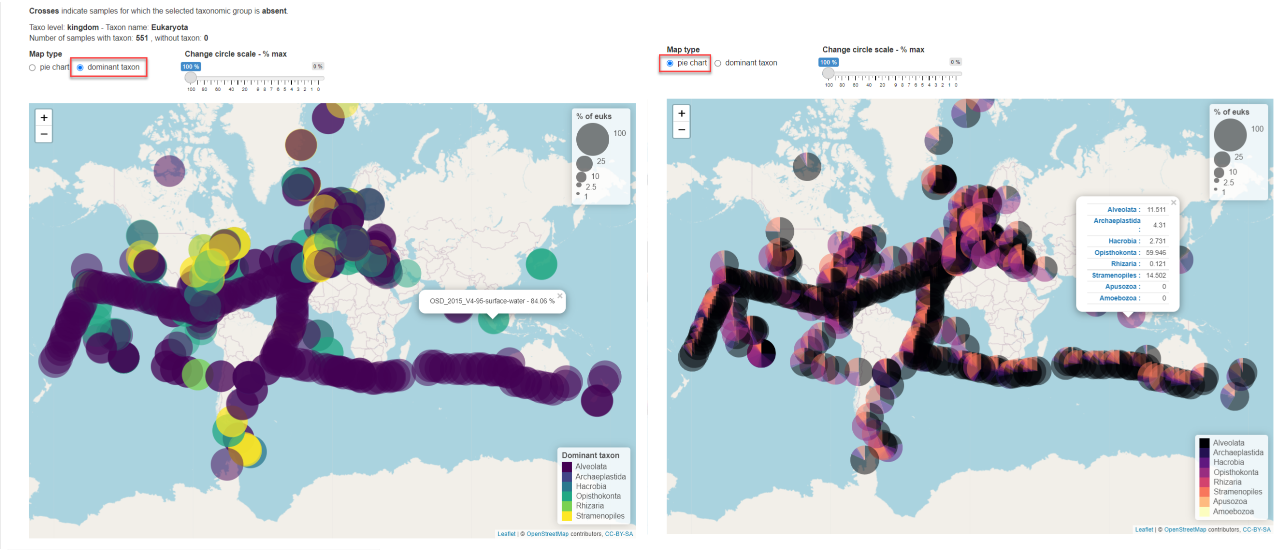

Figure S12: Shiny panel "Map".

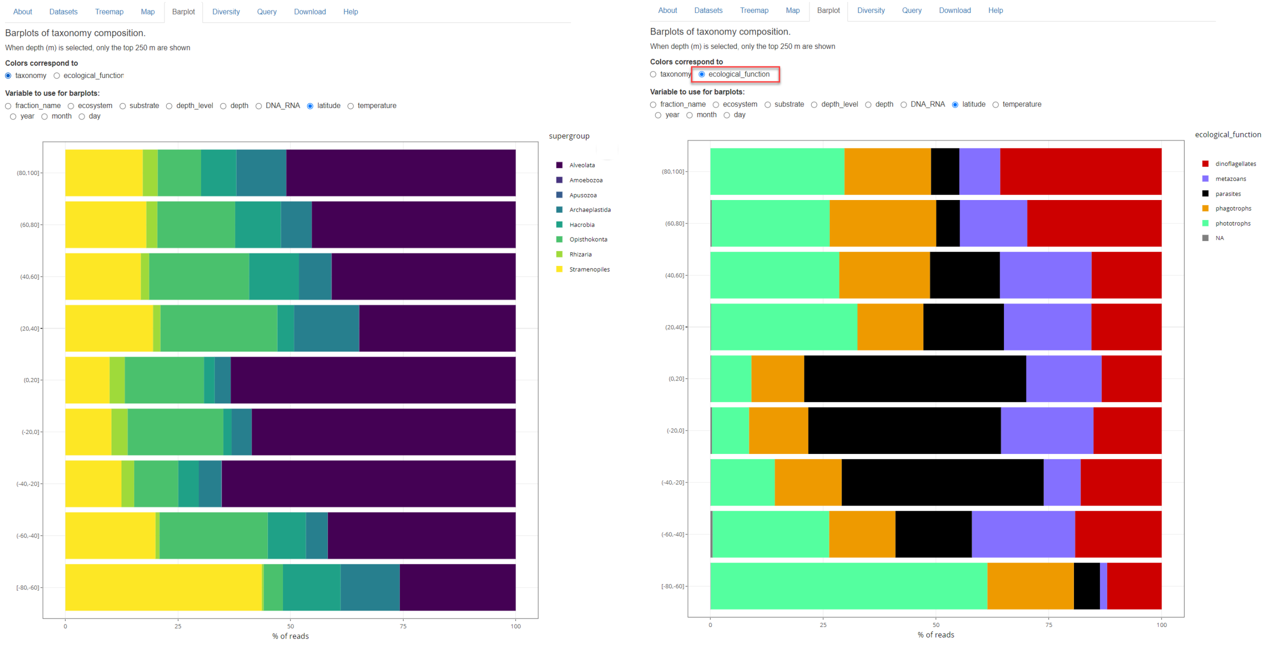

Figure S13: Shiny panel "Barplot".

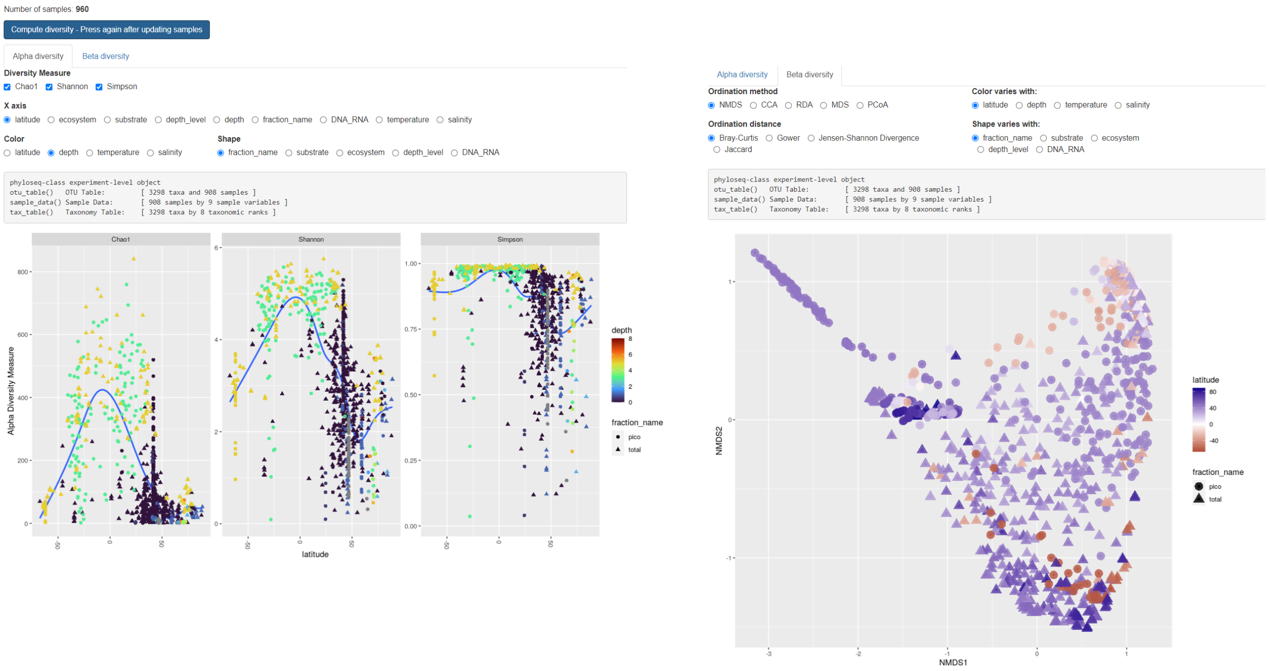

Figure S14: Shiny panel "Diversity".

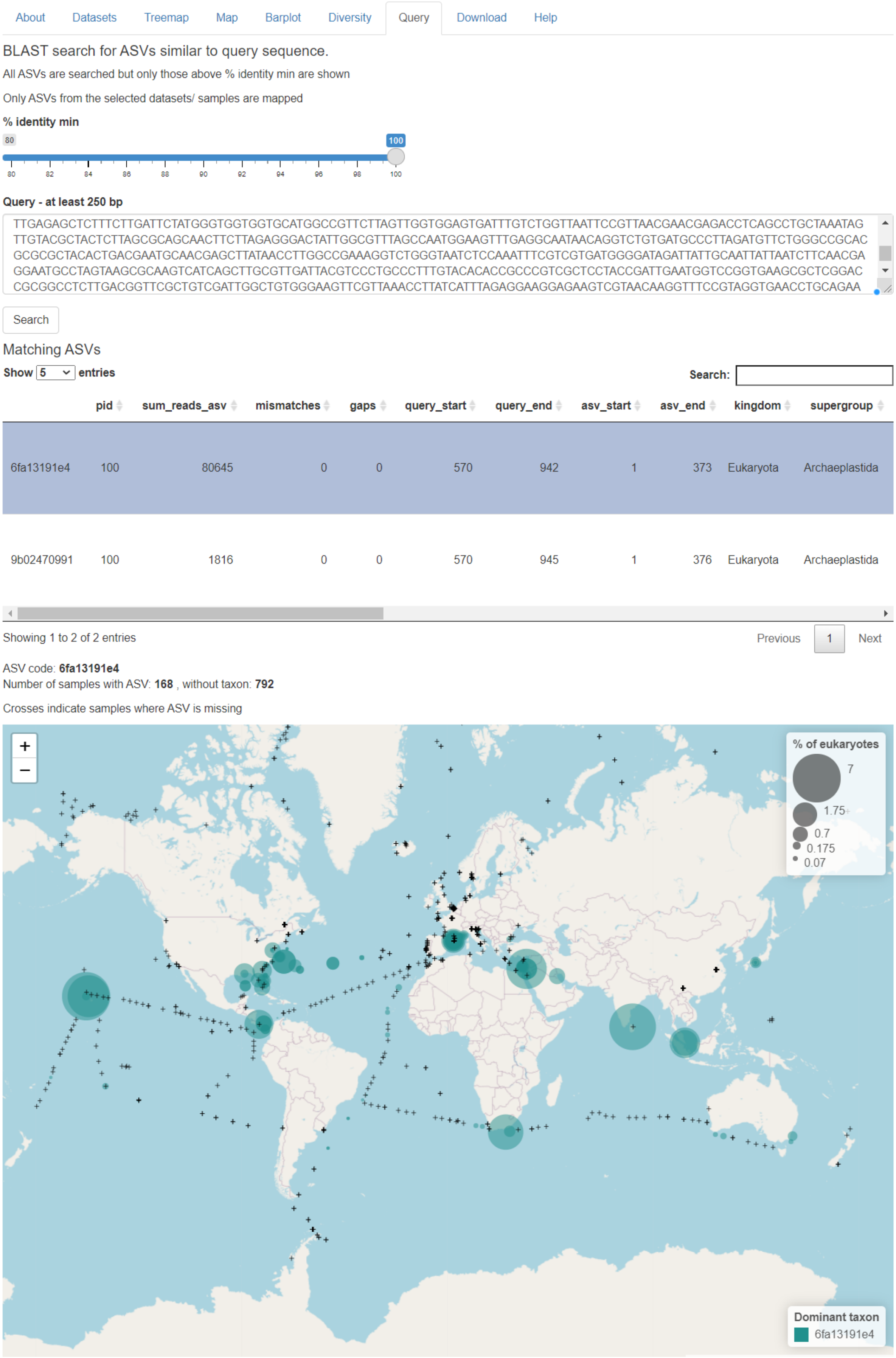

Figure S15: Shiny panel "Query".

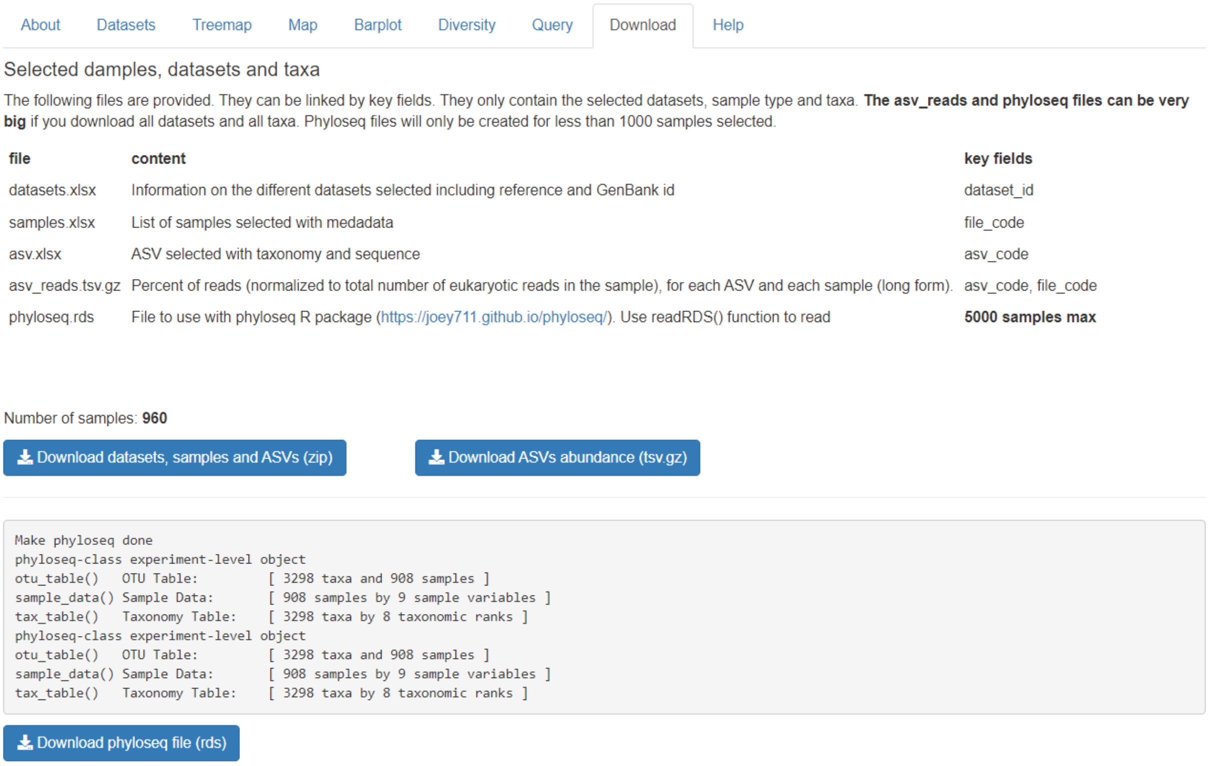

Figure S16: Shiny panel "Download".
